# Zinc homeostasis regulates caspase activity and inflammasome activation

**DOI:** 10.1371/journal.ppat.1012805

**Published:** 2024-12-17

**Authors:** Xiao Gong, Weidi Gu, Shuo Fu, Gonglu Zou, Zhengfan Jiang

**Affiliations:** 1 Key Laboratory of Cell Proliferation and Differentiation of the Ministry of Education, School of Life Sciences, Peking University, Beijing, China; 2 Peking-Tsinghua Center for Life Sciences, Peking University, Beijing, China; INSERM U851, FRANCE

## Abstract

Inflammasome activation drives pyroptotic cell death and the release of inflammatory cytokines, and many diseases involve its overactivation. Zinc is essential for all organisms as a trace element, but its functions in innate immunity remain undefined. Here, we reported that Zn^2+^ inhibits caspase-1 to hinder inflammasome activation. We first identified the zinc exporter solute carrier family 30 member 1 (SLC30A1) as an inflammasome regulator, using a genome-wide CRISPR-Cas9-mediated screen. *SLC30A1* deficiency suppressed multiple inflammasomes by increasing intracellular levels of Zn^2+^, which bound and inhibited caspase-1 at its active site residues H^237^, C^244^ and C^285^. Mutation of these residues almost completely blocked zinc binding. Similarly, Zn^2+^ also inhibited caspase-4/5/11-mediated noncanonical inflammasome activation. Importantly, zinc supplementation significantly relieved cecal ligation and puncture (CLP)-induced sepsis, Imiquimod (IMQ)-induced psoriasis and Alzheimer’s disease. Thus, zinc might be used to treat inflammasome-related diseases as a broad-spectrum inflammasome inhibitor.

## Author summary

Inflammasome overactivation causes cytokine release storm or autoimmune diseases, which threaten people’s health seriously. Here, we identified SLC30A1 as an important inflammasome regulator, the deficiency of which led to impaired activation of multiple inflammasomes. As the only zinc exporter transporting cytosolic zinc to the extracellular space, SLC30A1 deficiency increased intracellular zinc content, and Zn^2+^ bound inflammatory caspases to inhibit their activity directly. Furthermore, zinc supplementation alleviated inflammasome-related diseases, such as sepsis, psoriasis and Alzheimer’s disease. This finding provides a new clue for the prevention and treatment of inflammasome-related autoimmune diseases, and highlights the importance of zinc homeostasis in cells.

## Introduction

Inflammasomes are multiprotein complexes formed in cells responding to pathogen-associated molecular patterns (PAMPs) or damage-associated molecular patterns (DAMPs); they activate caspase-1, which promotes pyroptotic cell death (pyroptosis), as well as the maturation and secretion of IL-1β and IL-18 [1–4]. There are four major types of inflammasomes, including NLR family pyrin domain containing 1 (NLRP1), NLR family pyrin domain containing 3 (NLRP3), NLR family CARD domain containing 4 (NLRC4) and absent in melanoma 2 (AIM2). Among these inflammasomes, NLRP3 inflammasome is the most studied and activated by many stimulators, such as Nigericin, ATP, MSU and many pathogens [5,6]. Upon stimulation, NLRP3 oligomerizes with NEK7 [7–10] and interacts with the adaptor protein apoptosis-associated speck-like protein containing a CARD (ASC) [11]; ASC then assembles to recruit caspase-1 [12,13], leading to caspase-1 activation and gasdermin D (GSDMD)-mediated pyroptosis [2,14–16]. Abnormal inflammasome activation is involved in many inflammatory, autoimmune, metabolic, neurologic diseases and cancer [17–20]. Therefore, it is of great importance to identify effective inflammasome inhibitors to treat these diseases.

Zinc is the second most abundant trace element in the body after Fe [21] and a well-recognized cytoprotectant due to its capacity to minimize oxidative damage [22,23]. It exists as a divalent cation (Zn^2+^) and is essential in many biological processes for all organisms. Zn transporters (ZnTs) and Zrt/Irt-related proteins (ZIPs) function in zinc influx, efflux and compartmentalization across biological membranes [21] to control zinc homeostasis in cells. Zinc deficiency in humans causes immune insufficiency and chronic inflammation. Previous work showed that zinc inhibits apoptotic caspases to regulate apoptosis [24,25] and intracellular zinc depletion even induces caspase activation and IL-1β secretion [26,27]. Pyroptosis is another type of programmed cell death mediated by inflammatory caspases, in which the role of zinc has not been reported.

In this study, using a genome-wide CRISPR-Cas9-mediated screen and biochemical analysis, we identified *SLC30A1* had an important role in regulating multiple inflammasome activation. *SLC30A1* deficiency increased intracellular zinc content significantly, and Zn^2+^ bound inflammatory caspases to inhibit their activity directly, causing impaired inflammasome activation. Importantly, zinc supplementation weakened inflammation *in vivo* thus alleviating autoimmune diseases including sepsis, psoriasis and Alzheimer’s disease. Our results provide a new strategy for the prevention and treatment of autoimmune diseases caused by dysregulated inflammasome activation.

## Results

### Identification of *SLC30A1* by a genome-wide CRISPR-Cas9-mediated screen

**Fig 1:**
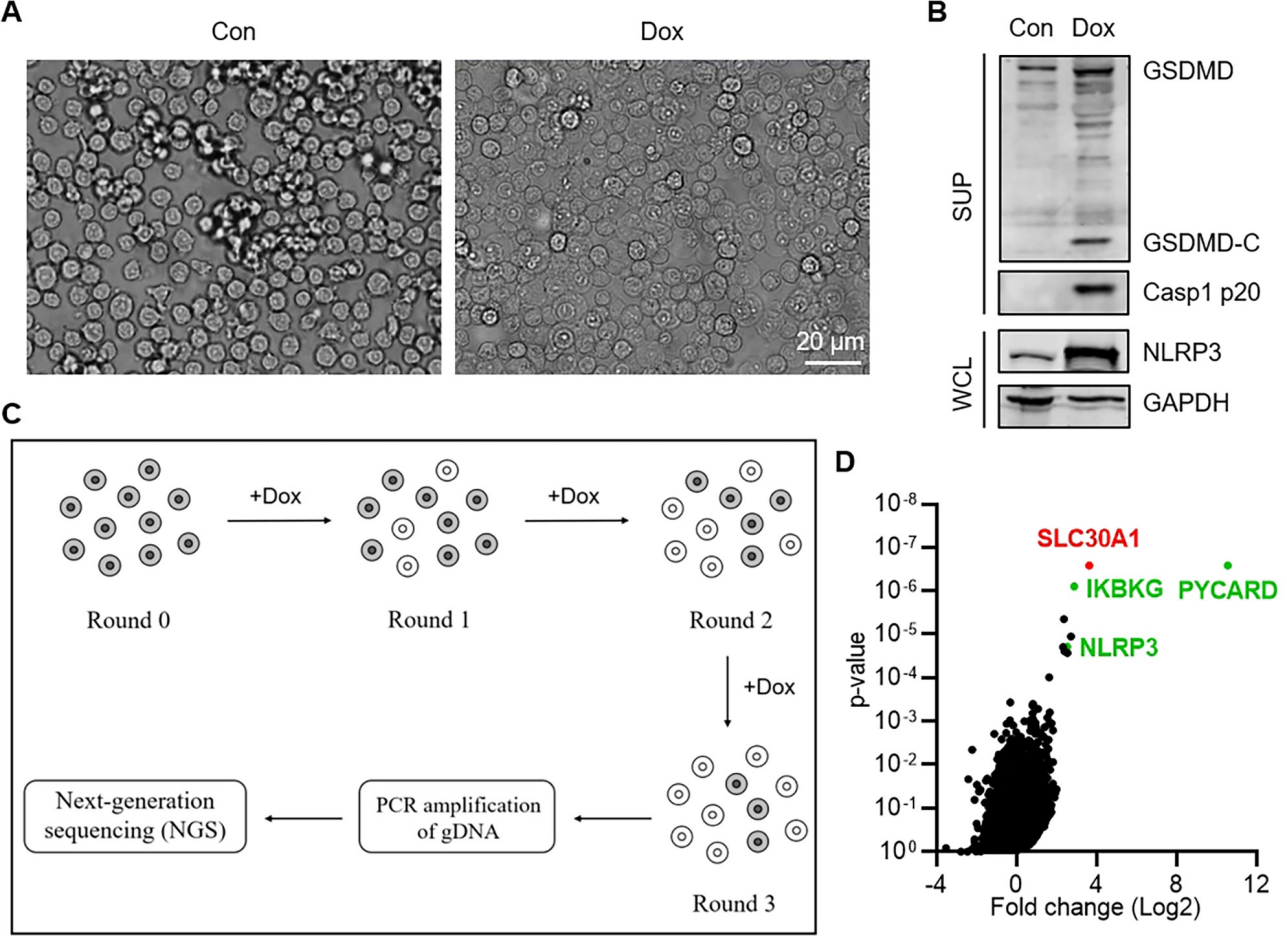
Identification of SLC30A1 by a genome-wide CRISPR-Cas9-mediated screen. (A) Microscopy of pTRE3G-NLRP3 Tet-on THP-1 cells before and after Dox treatment (100 ng/mL, 8 h). Scale bar, 20 μm. (B) Immunoblotting analysis of the indicated proteins in pTRE3G-NLRP3 Tet-on THP-1 cells treated with Dox (100 ng/mL) for 8 h. (C) Schematic illustration of the genome-wide CRISPR-Cas9-mediated screen. pTRE3G-NLRP3 Tet-on THP-1 cells stably expressing a genome-wide sgRNA library and Cas9 (Round 0) were subjected to three rounds (Round 1-Round 3) of selection. Enriched genes in the surviving cells (Round 3) were identified by next-generation sequencing (NGS) analysis. (D) Genes identified by the screen were ranked and plotted. The x axis shows the fold change of genes (Round 3/Round 0). The y axis shows the p-value. Genes ranking in the front that involved in NLRP3 inflammasome pathway were plotted with green dots. *SLC30A1*, was plotted with a red dot.

To search for key genes involved in inflammasome activation, we set up a Tet-on gene expression system to control NLRP3 expression in cells. A THP-1 stably expressing pTRE3G-NLRP3 Tet-on cell line was first constructed, which showed a nice response to Dox with apparent NLRP3 activation and pyroptosis (Fig 1A and 1B), in a Dox concentration-dependent manner (S1A and S1B Fig). The autoactivation of NLRP3 was due to its overexpression, so there was no IL-1β and less TNF-α release because of no LPS induction (S1C and S1D Fig). Next, a genome-wide CRISPR-Cas9-mediated screen was carried out using this cell line. After three rounds of selection, genes targeted by sgRNAs in surviving cells were identified by next-generation sequencing (NGS) analysis and ranked based on the number of unique sgRNAs (Fig 1C), which showed enriched genes in NLRP3 inflammasome signaling pathway, such as *PYCARD*, *IKBKG* and *NLRP3* (Fig 1D), indicative of a successful screen. Meanwhile, *SLC30A1*, which encodes SLC30A1 or Zinc transporter 1 (ZnT1) [28] was significantly enriched with all four sgRNAs (S1E Fig).

### *SLC30A1* deficiency suppresses inflammasome activation

**Fig 2:**
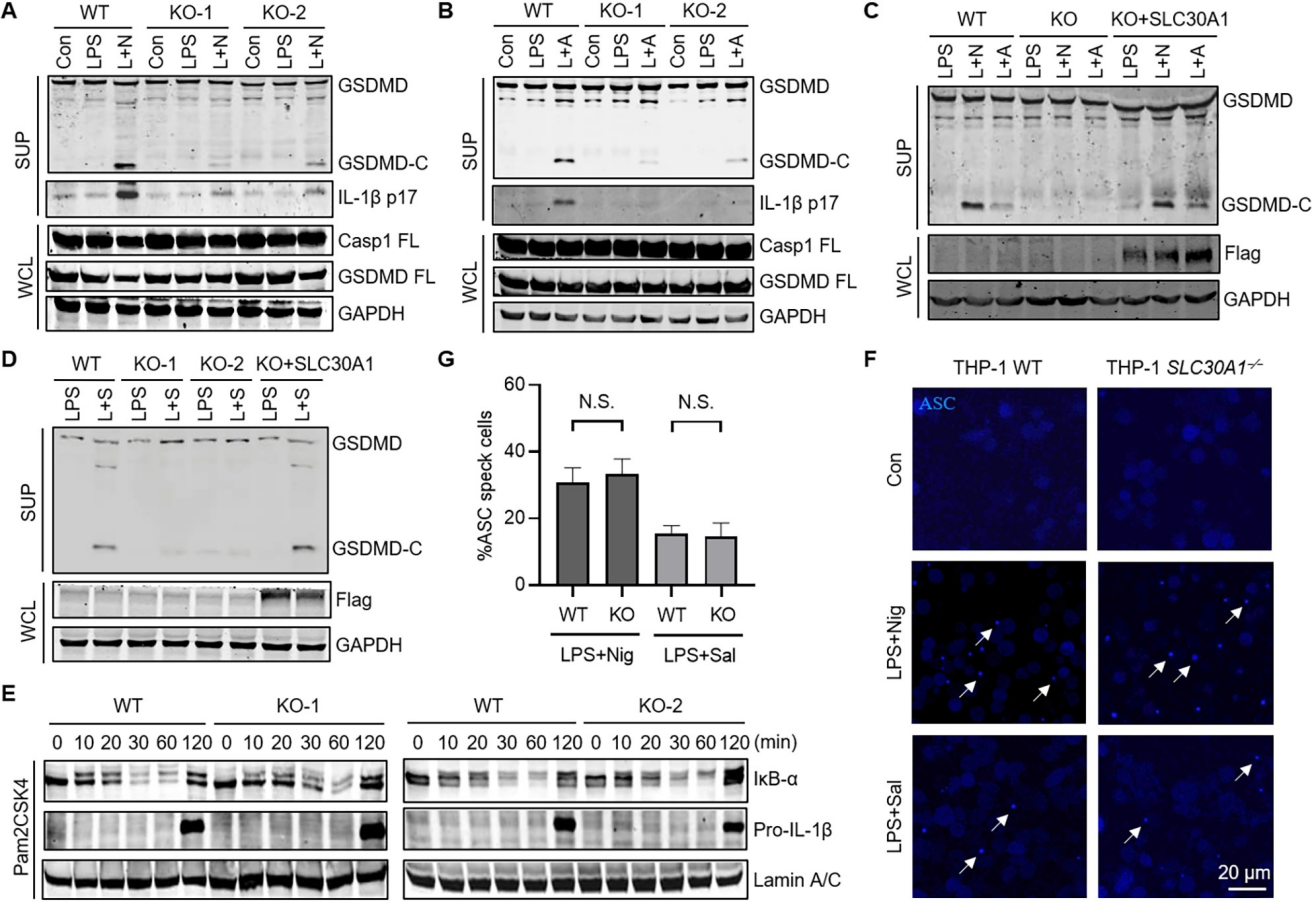
*SLC30A1* deficiency suppresses inflammasome activation. (A and B) Immunoblotting analysis of the indicated proteins in WT and *SLC30A1*^*−/−*^ THP-1 cells pretreated with LPS (1 μg/mL) for 3 h, followed by Nigericin (2.5 μM) (A) for 1 h or ATP (2.5 mM) (B) for 3 h. L + N, LPS + Nig; L + A, LPS + ATP. (C) Immunoblotting analysis of the indicated proteins in WT, *SLC30A1*^*−/−*^ and SLC30A1 re-expression THP-1 cells pretreated with LPS (1 μg/mL) for 3 h, followed by Nigericin (2.5 μM) for 1 h or ATP (2.5 mM) for 3 h. L + N, LPS + Nig; L + A, LPS + ATP. (D) Immunoblotting analysis of the indicated proteins in WT, *SLC30A1*^*−/−*^ and SLC30A1 re-expression THP-1 cells pretreated with LPS (1 μg/mL) for 3 h, followed by *Salmonella Typhimurium* (MOI = 5) for 1 h. L + S, LPS + *S*. *Typhimurium*. (E) Immunoblotting analysis of the indicated proteins in WT and *SLC30A1*^*−/−*^ THP-1 cells treated with Pam2CSK4 (10 ng/mL) for the indicated times. (F) Fluorescence microscopy of WT and *SLC30A1*^−/−^ THP-1 cells stably expressing BFP-ASC pretreated with LPS (1 μg/mL) for 3 h, followed by Nigericin (2.5 μM) for 1 h or *Salmonella Typhimurium* (MOI = 5) for 1 h. ASC specks were pointed by white arrows. Scale bar, 20 μm. (G) Quantification of percentage of WT and *SLC30A1*^−/−^ THP-1 cells stably expressing BFP-ASC containing ASC speck after the indicated treatment. Data are the mean ± SD. Student’s t-test was used to analyze data. N.S., not significant.

To test whether SLC30A1 plays any roles in NLRP3 activation, *SLC30A1* was first deleted in THP-1 cells, confirmed by immunoblotting and sequencing (S2A and S2B Fig). Consistent with its enrichment in the screen, *SLC30A1* deficiency in THP-1 cells impaired NLRP3 inflammasome activation by Nigericin or ATP treatment (Fig 2A and 2B), and re-expression of SLC30A1 in *SLC30A1*^*−/−*^ THP-1 cells restored NLRP3 activation (Fig 2C). Moreover, *SLC30A1* deficiency similarly abolished GSDMD cleavage induced by *Salmonella Typhimurium* infection, which activates NLRC4 inflammasome [29] (Fig 2D), indicating that *SLC30A1* is also important for other inflammasomes. *SLC30A1*^*−/−*^ THP-1 cells showed normal NF-κB activation (Fig 2E) and ASC speck formation in response to different stimuli (Fig 2F and 2G). In addition, *SLC30A1* deficiency enhanced DNA virus-induced innate immune responses (S2C and S2D Fig), consistent with previous reports that inflammasome activation negatively regulates the cGAS-STING pathway [30–32].

### *SLC30A1* deficiency increases intracellular zinc content

**Fig 3:**
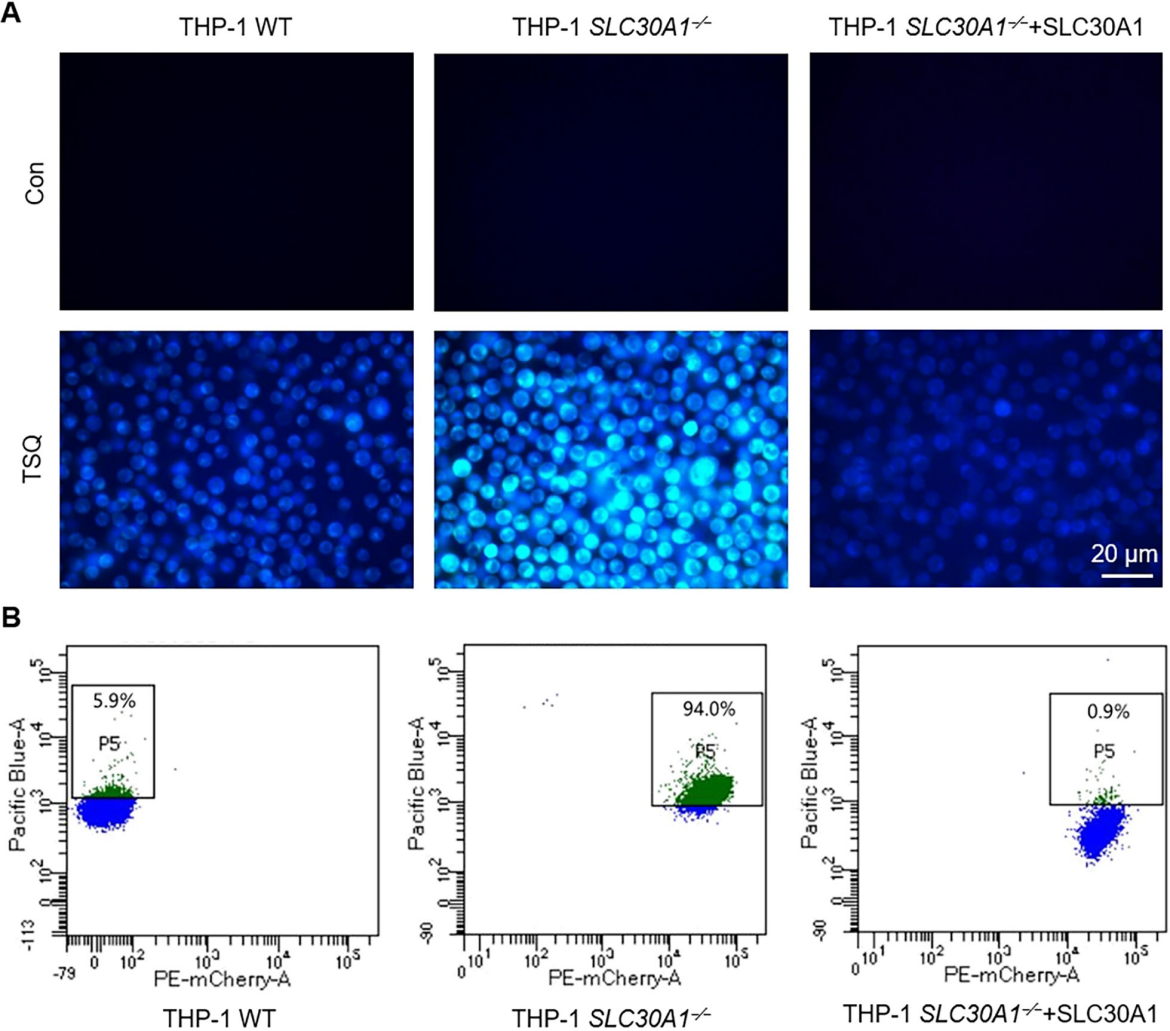
*SLC30A1* deficiency increases intracellular zinc content. (A) Fluorescence microscopy of WT, *SLC30A1*^*−/−*^ and SLC30A1 re-expression THP-1 cells treated with TSQ (5 μg/mL) for 30 min. The intracellular zinc content is characterized by blue fluorescence. Scale bar, 20 μm. (B) Flow cytometry analysis of WT, *SLC30A1*^*−/−*^ and SLC30A1 re-expression THP-1 cells treated with TSQ (5 μg/mL) for 30 min. P5 represents positive cell population.

ZnT1 is the only zinc transporter located on the plasma membrane to export cytosolic zinc to the extracellular space with the rest of ZnTs (ZnT2-ZnT10 encoded by *SLC30A2-SLC30A10*) transporting zinc into the luminal sides of organelles [21,33], so we used TSQ (N-(6-methoxy-8-quinolyl)-p-toluenesulfonamide) (S3A Fig), a neutral pH fluorescent probe for zinc, to detect zinc content in ZnT1-deficient cells. Indeed, *SLC30A1*^*−/−*^ THP-1 cells showed significantly increased levels of zinc compared to the wildtype (WT) THP-1 cells, and this effect was reversed by the re-expression of SLC30A1 in *SLC30A1*^*−/−*^ THP-1 cells (Figs 3A and S3B). Similar results were obtained by flow cytometry analysis and ICP-MS (Figs 3B and S3C). Therefore, *SLC30A1* deficiency resulted in increased intracellular zinc level as zinc cannot be transported out of cells.

### Zinc inhibits inflammasome activation by suppressing caspase-1

Since the intracellular zinc level in *SLC30A1*^*−/−*^ THP-1 cells increased significantly, it is possible that zinc affects inflammasome activation, as zinc was found to inhibit apoptotic caspases. We treated pTRE3G-NLRP3 Tet-on THP-1 cells with Dox and different divalent metal cations, and found that Mn^2+^ enhanced NLRP3 activation, consistent with previous reports [34,35], while Zn^2+^ effectively suppressed NLRP3 activation (S4A Fig).

**Fig 4:**
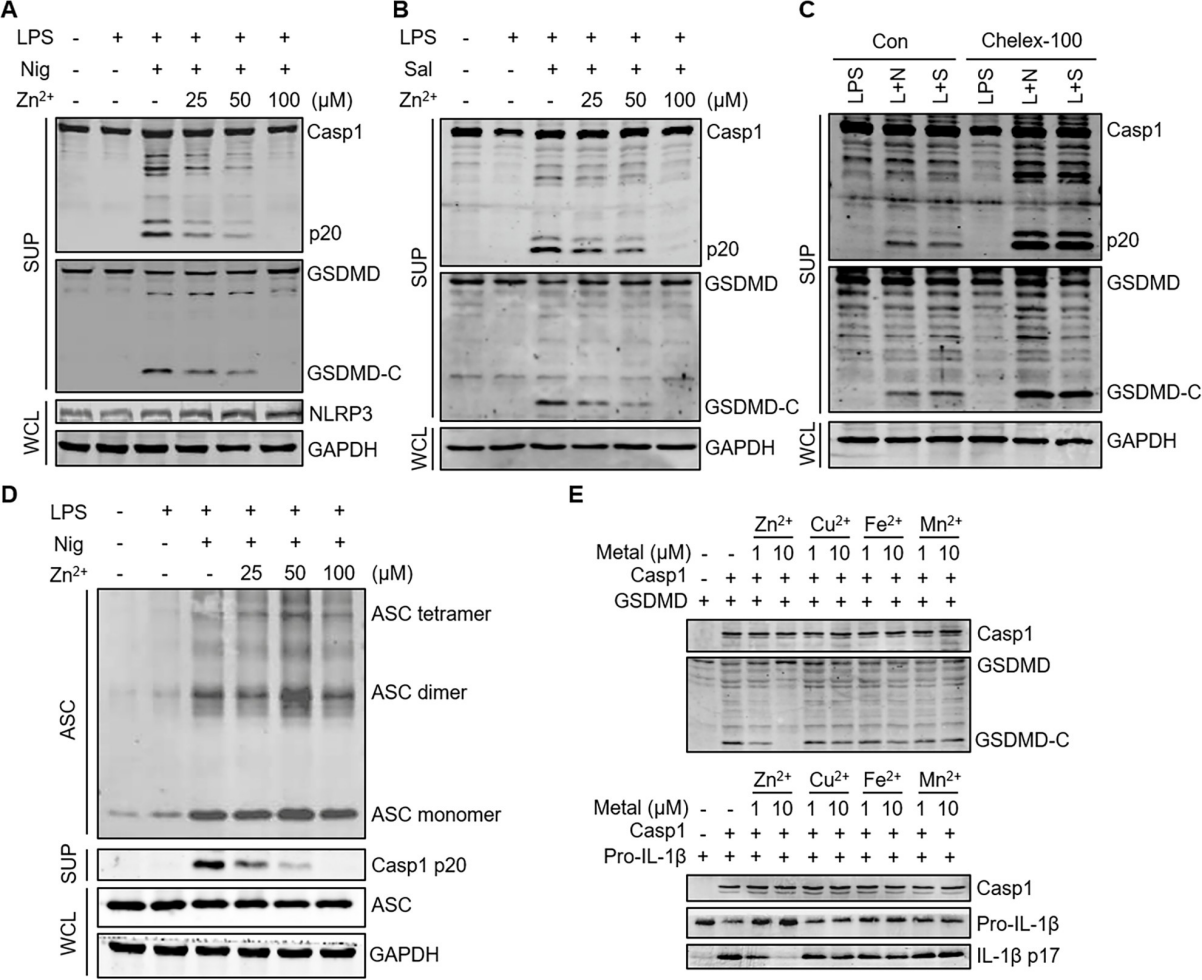
Zinc inhibits inflammasome activation by suppressing caspase-1. (A and D) Immunoblotting analysis of the indicated proteins in WT THP-1 cells pretreated with LPS (1 μg/mL) for 3 h and the indicated concentration of ZnCl_2_ for 1 h in turn, followed by Nigericin (5 μM) for 1 h. (B) Immunoblotting analysis of the indicated proteins in WT THP-1 cells pretreated with LPS (1 μg/mL) for 3 h and the indicated concentration of ZnCl_2_ for 1 h in turn, followed by *Salmonella Typhimurium* (MOI = 10) for 1 h. (C) Immunoblotting analysis of the indicated proteins in WT THP-1 cells and Chelex-100-treated WT THP-1 cells pretreated with LPS (1 μg/mL) for 3 h, followed by Nigericin (5 μM) for 1 h or *Salmonella Typhimurium* (MOI = 10) for 1 h. L + N, LPS + Nig; L + S, LPS + *S*. *Typhimurium*. (E) Immunoblotting analysis of the indicated protein from *in vitro* cleavage assay with 250 ng caspase-1, 1 μg GSDMD or pro-IL-1β and the indicated concentration of ZnCl_2_, CuCl_2_, FeCl_2_ or MnCl_2_ at 37°C for 2 h.

To confirm this observation, we treated THP-1 cells with different concentrations of zinc chloride and found that NLRP3 activation was suppressed in a Zn^2+^ concentration-dependent manner, while the expression of NLRP3 was unaffected (Fig 4A). Similarly, NLRC4 activation was inhibited dependent on Zn^2+^ concentrations (Fig 4B). In contrast, the activation of both NLRP3 and NLRC4 inflammasomes was significantly enhanced in cells treated by the Zn^2+^ chelating resin Chelex-100 [36] to deprive zinc in the culture medium (Fig 4C). As Zn^2+^ inhibited multiple inflammasomes, we reasoned that Zn^2+^ executes on common protein(s) in inflammasome pathways. THP-1 cells showed normal IκB-α degradation and pro-IL-1β induction in response to LPS after the treatment of Zn^2+^ below 200 μM (S4B Fig), and the maximum Zn^2+^ concentration used in this study was 100 μM. Thus, the NF-κB pathway in these cells was unlikely affected. Then, the formation of ASC oligomer was determined since ASC must be oligomerized to transmit activating signals to its downstream component caspase-1 [12,13]. It was found that ASC oligomerization was unaffected by increasing Zn^2+^ concentration, while the activation of caspase-1 was completely abolished (Fig 4D). These results indicated that Zn^2+^ works on caspase-1, instead of ASC. To prove this, bacterially purified caspase-1 and GSDMD or pro-IL-1β were incubated in the presence of different divalent metal cations to perform an *in vitro* caspase cleavage assay, followed by immunoblotting. The cleavage of GSDMD and pro-IL-1β by caspase-1 was clearly inhibited by Zn^2+^, but not by other divalent metal cations (Fig 4E). These results demonstrated that Zn^2+^ inhibits inflammasome activation by impeding the activity of caspase-1.

### Zinc binds inflammatory caspases at a conserved His-Cys-Cys triad

**Fig 5:**
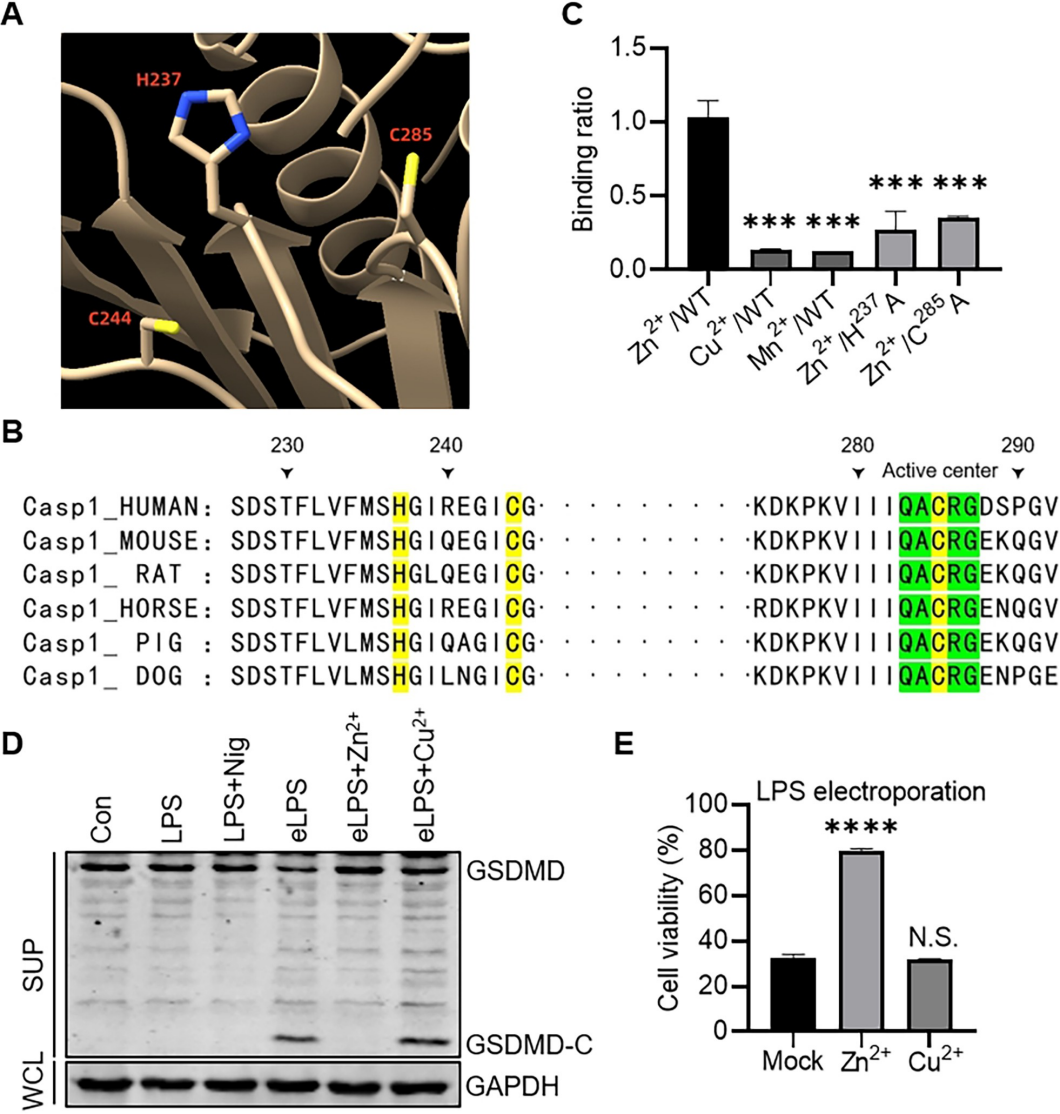
Zinc binds inflammatory caspases at a conserved His-Cys-Cys triad. (A) Zn^2+^-binding site of caspase-1. The protein structure data was downloaded from PDB (1ICE) and opened by ChimeraX. (B) Sequence alignment of caspase-1 from different species. Protein sequences were obtained from UniProt. Casp1_HUMAN: P29466; Casp1_MOUSE: P29452; Casp1_RAT: P43527; Casp1_HORSE: Q9TV13; Casp1_PIG: Q9N2I1; Casp1_DOG: Q9MZV7. (C) Zn^2+^/Cu^2+^/Mn^2+^-binding stoichiometry of caspase-1 and variants identified by ICP-MS. ANOVA test was used to analyze data. ***p < 0.001. (D) Immunoblotting analysis of the indicated proteins in *CASP1*^*−/−*^ THP-1 cells pretreated with ZnCl_2_ (50 μM) or CuCl_2_ (50 μM) for 1 h, followed by LPS (1 μg/10^6^ cells) electroporation. (E) Cell viability analysis of *CASP1*^*−/−*^ THP-1 cells pretreated with ZnCl_2_ (50 μM) or CuCl_2_ (50 μM) for 1 h, followed by LPS (1 μg/10^6^ cells) electroporation. Data are the mean ± SD. ANOVA test was used to analyze data. N.S., not significant, p > 0.05; ****p < 0.0001.

Human caspase-1 consists of 404 amino acids, which can be divided into an N-terminal CARD domain, an intermediate p20 subunit and a C-terminal p10 subunit. After activation, caspase-1 is self-cleaved at D^103^, D^119^, D^297^ and D^316^ to generate p20 and p10 that form p20/p10 heterodimer. Then, two p20/p10 heterodimers form a homodimer through the polymerization of p10 subunits to complete the activation (S5A Fig). *In vitro* assay using bacterially purified caspases showed that Zn^2+^ inhibited apoptotic caspases, in which caspase-6 was completely inhibited by 0.1 mM Zn^2+^ and caspase-3, -7 or -8 by approximately 1 mM Zn^2+^ [25]. Previous studies indicated that the binding of Zn^2+^ to caspases usually requires three amino acids including one histidine and two cysteine residues, as Zn^2+^ binds caspase-9 at the active site composed of H^237^, C^239^, C^287^ and an exosite composed of H^224^, C^230^, and C^272^ [37] (S5B Fig). Indeed, we confirmed that Zn^2+^ potently prevented ABT-263-induced apoptosis (S5C Fig) as previous work demonstrated [24,25]. Caspase-1 contains eight histidine residues, among which only H^237^ is surrounded by two cysteines C^244^ and C^285^ (Fig 5A) to make a nice His-Cys-Cys triad for zinc binding [37]. Importantly, H^237^, C^244^ and C^285^ are conserved among different species (Fig 5B) and essential for its activity, as mutating each of these residues eliminated pro-IL-Iβ processing activity as well as its autoprocessing [38]. We found that, by ICP-MS, each caspase-1 bound one Zn^2+^ instead of other divalent metal cations, and mutation of either H^237^ or C^285^ almost completely blocked Zn^2+^ binding (Fig 5C). A colorimetric zinc indicator Zincon [39] was used to determine Zn^2+^-binding stoichiometry by titrating Zn^2+^ into samples containing zincon and caspase-1, from which the same conclusion was obtained as by ICP-MS (S5D Fig). In addition, caspase-4/5 (human) and caspase-11 (mouse) also have conserved His-Cys-Cys residues around the active site similar to caspase-1 (S5E Fig), indicating that Zn^2+^ may also inhibit caspase-4/5/11-mediated noncanonical inflammasome activation. To test this, LPS was electroporated into *CASP1*^*−/−*^ THP-1 cells using the Neon Transfection System. The cleavage of GSDMD and pyroptosis were clearly inhibited by Zn^2+^, but not by other divalent cations like Cu^2+^ (Fig 5D and 5E).

### Zinc inhibits inflammatory response *in vivo*

**Fig 6:**
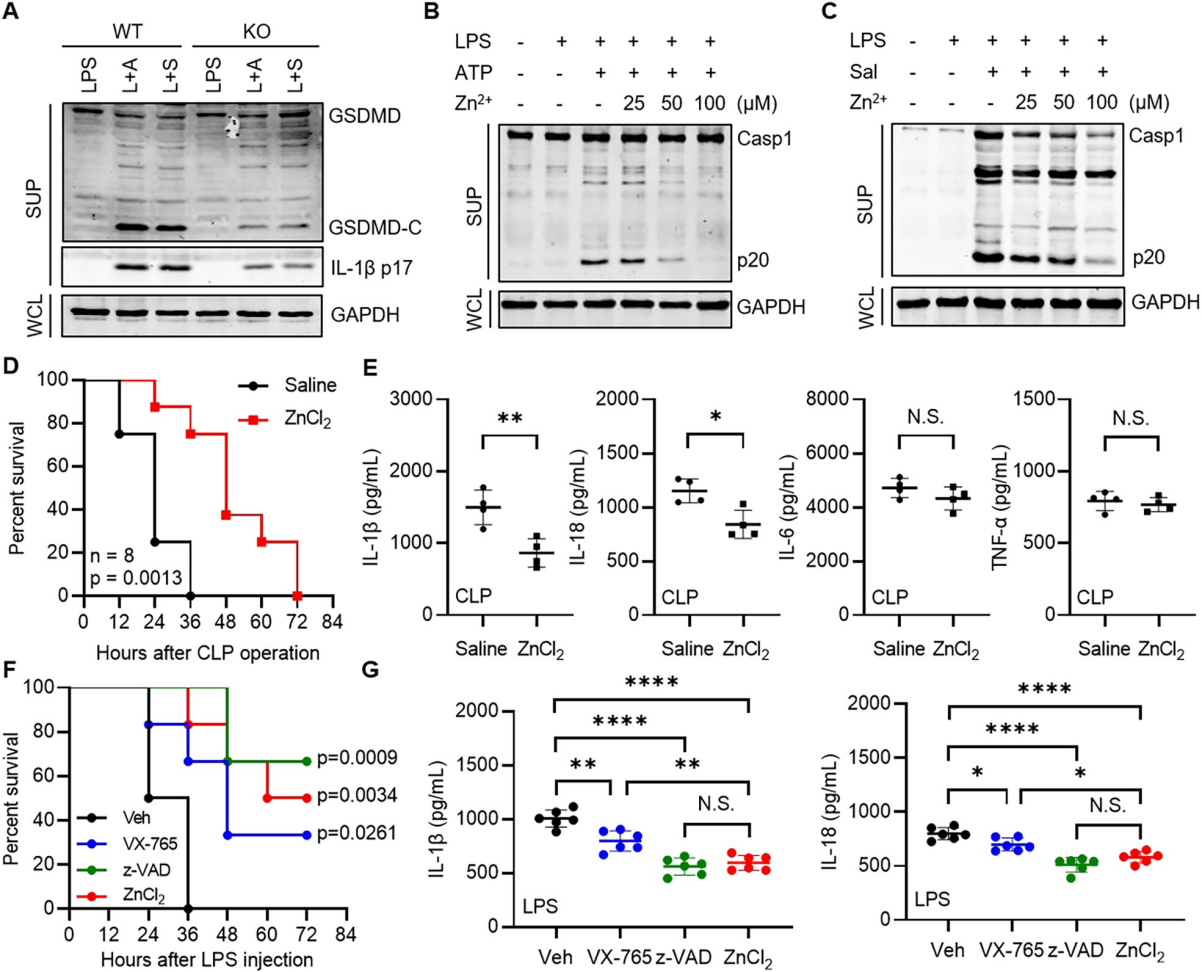
Zinc inhibits inflammatory response *in vivo*. (A) Immunoblotting analysis of the indicated proteins in WT and *Slc30a1*^*−/−*^ iBMDM cells pretreated with LPS (1 μg/mL) for 3 h, followed by ATP (2.5 mM) for 3 h or *Salmonella Typhimurium* (MOI = 5) for 1 h. L + A, LPS + ATP; L + S, LPS + *S*. *Typhimurium*. (B and C) Immunoblotting analysis of the indicated proteins in WT iBMDM cells pretreated with LPS (1 μg/mL) for 3 h and the indicated concentration of ZnCl_2_ for 1 h in turn, followed by ATP (5 mM) (B) for 3 h or *Salmonella Typhimurium* (MOI = 10) (C) for 1 h. (D) Survival of the mice (n = 8) pretreated (i.n.) with saline or ZnCl_2_ (2 mg Zn/kg) for 24 h, followed by cecal ligation and puncture operation. Survival curves were compared using Mantel-Cox test. (E) IL-1β, IL-18, IL-6 and TNF-α concentration of sera from the mice pretreated (i.n.) with saline or ZnCl_2_ (2 mg Zn/kg) for 24 h, followed by cecal ligation and puncture operation. Blood samples were collected at 6 h after the operation. Data are the mean ± SD (n = 4). Student’s t-test was used to analyze data. N.S., not significant, p > 0.05; *p < 0.05; **p < 0.01. (F) Survival of the mice (n = 6) pretreated (i.p.) with vehicle (5% DMSO + 95% saline), VX-765 (25 mg/kg), z-VAD-FMK (25 mg/kg) or ZnCl_2_ (2 mg Zn/kg) for 24 h, followed by LPS (30 mg/kg) injection (i.p.). Survival curves were compared using Mantel-Cox test. (G) IL-1β and IL-18 concentration of sera from the mice pretreated (i.p.) with vehicle (5% DMSO + 95% saline), VX-765 (25 mg/kg), z-VAD-FMK (25 mg/kg) or ZnCl_2_ (2 mg Zn/kg) for 24 h, followed by LPS (30 mg/kg) injection (i.p.). Blood samples were collected at 12 h after the injection. Data are the mean ± SD (n = 6). ANOVA test was used to analyze data. *p < 0.05; **p < 0.01; ****p < 0.0001.

To investigate whether these results hold true for murine cells, we deleted *Slc30a1* in iBMDM (immortalized bone marrow-derived macrophages) cells, which was confirmed by sequencing (S6A Fig). Similar to THP-1 cells, *Slc30a1* deficiency increased intracellular zinc content significantly in iBMDM cells (S6B–S6E Fig). As expected, NLRP3 and NLRC4 inflammasomes were both suppressed in *Slc30a1*^*−/−*^ iBMDM cells after ATP treatment or *Salmonella Typhimurium* infection (Fig 6A). Consistently, the activation of NLRP3 and NLRC4 in wildtype (WT) iBMDM cells was inhibited by Zn^2+^ in a concentration-dependent manner (Fig 6B and 6C). These results proved that zinc suppressed inflammasomes in both human and mouse cells.

Next, we explored the physiological function of zinc in inflammatory response *in vivo*. Excessive secretion of inflammatory cytokines causes sepsis. The mouse model of cecal ligation and puncture (CLP), which has been used extensively to investigate sepsis and septic shock, was utilized. Due to the features of high-absorption, non-invasive and no-damage [40,41], we chose intranasal administration of zinc chloride (2 mg Zn/kg) to mice. It was found that intranasal Zn^2+^-pretreatment before CLP significantly prolonged the survival of mice (Fig 6D), which was slightly better than intravenous Zn^2+^-pretreatment (S6F Fig). Moreover, ELISA analysis of cytokines in sera revealed that Zn^2+^-pretreated mice produced much less IL-1β and IL-18 than control mice, while the levels of other cytokines like IL-6 and TNF-α were comparable in sera (Fig 6E). Interestingly, we compared the effect of zinc supplementation in inhibiting inflammation with VX-765 (a caspase-1-specific inhibitor) and z-VAD-FMK (a pan-caspase inhibitor), and found that zinc was more effective than VX-765 in reducing LPS-induced death (Fig 6F) and IL-1β/18 production (Fig 6G) in mice, similar to z-VAD-FMK, suggesting that zinc inhibits both inflammatory and apoptotic cell death *in vivo* and that acute apoptotic cell death contributes to the death of mouse probably by inducing necroptosis and tissue/organ damages. These results collectively indicated that zinc inhibits inflammatory response *in vivo*.

### Zinc homeostasis regulates autoimmune diseases

**Fig 7:**
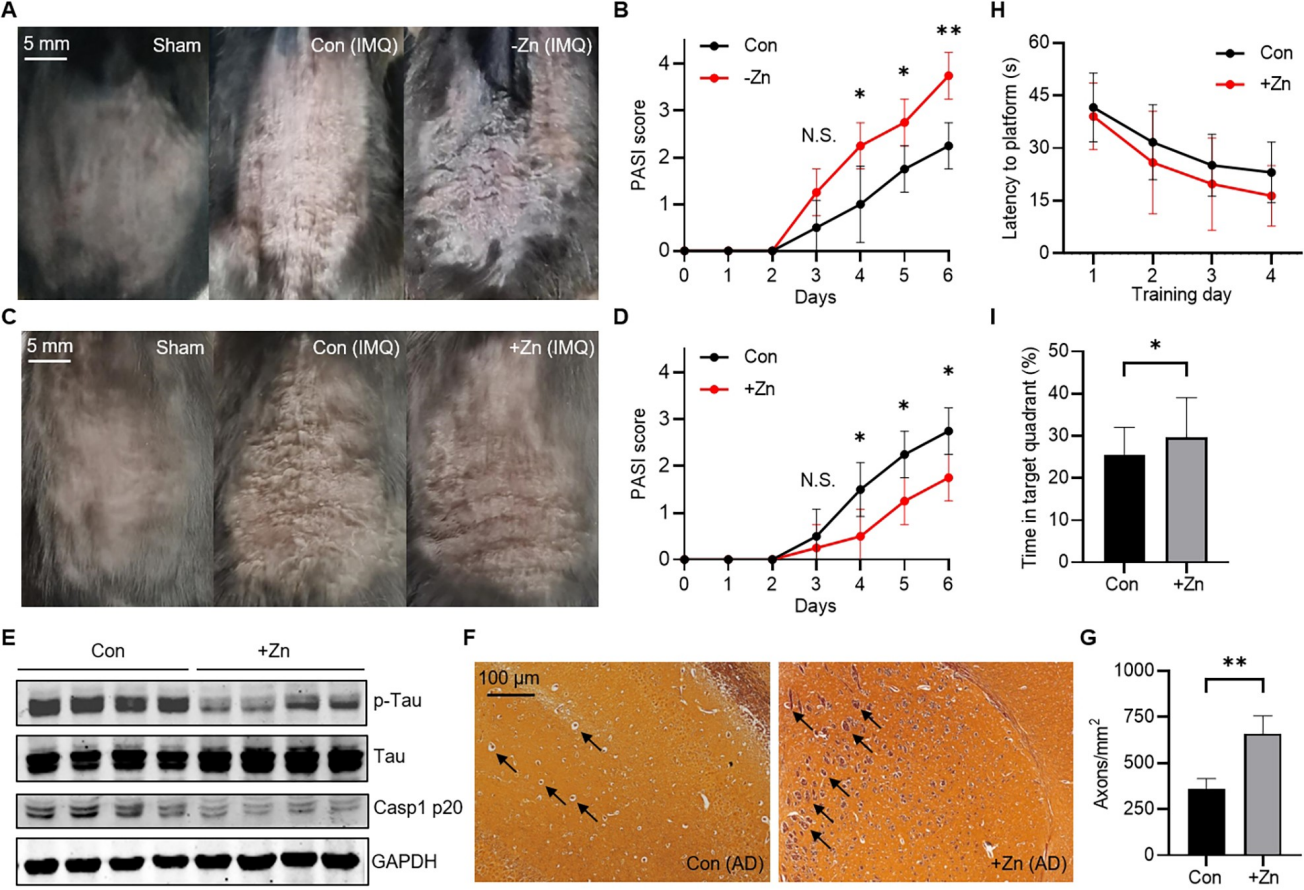
Zinc homeostasis regulates autoimmune diseases. (A) Photographs of the control (Con) and Zn-insufficient (-Zn) psoriasis mice with scaling skin. Scale bar, 5 mm. (B) PASI score of the control (Con) and Zn-insufficient (-Zn) psoriasis mice. Higher score indicates severer symptoms. Data are the mean ± SD (n = 4). Student’s t-test was used to analyze data. N.S., not significant, p > 0.05; *p < 0.05; **p < 0.01. (C) Photographs of the control (Con) and Zn-rich (+Zn) psoriasis mice with scaling skin. Scale bar, 5 mm. (D) PASI score of the control (Con) and Zn-rich (+Zn) psoriasis mice. Higher score indicates severer symptoms. Data are the mean ± SD (n = 4). Student’s t-test was used to analyze data. N.S., not significant, p > 0.05; *p < 0.05. (E) Immunoblotting analysis of the indicated proteins in the brain from the control (Con) and Zn-rich (+Zn) APP/PS1 mice (n = 4). (F) Glycine silver staining for the brain from the control (Con) and Zn-rich (+Zn) APP/PS1 mice. The axons were pointed by black arrows. Scale bar, 100 μm. (G) Axonal density of brains from the control (Con) and Zn-rich (+Zn) APP/PS1 mice. Data are the mean ± SD (n = 3). Student’s t-test was used to analyze data. **p < 0.01. (H and I) Quantification of latency to platform during the training phase (H) and percentage of time spent in the target quadrant during the probe test (I) by Morris water maze test of the control (Con) and Zn-rich (+Zn) APP/PS1 mice. Data are the mean ± SD (n = 6). Student’s t-test was used to analyze data. *p < 0.05.

Finally, we investigated the effect of zinc homeostasis on two autoimmune diseases. To begin with, we established a Zn-insufficient mouse model using six weeks old C57BL/6J mice. Two groups of mice were fed *ad libitum* with the standard diet (140 mg Zn/kg) or Zn-insufficient diet (lower than 5 mg Zn/kg) for six weeks. During this period of feeding, Zn-insufficient mice lived in a good healthy condition (S7A Fig), but zinc content in the various organs decreased significantly while the content of other trace elements like Cu and Mn remained unchanged (S7B and S7C Fig). Next, we used Imiquimod (IMQ) cream to induce psoriasis in these Zn-insufficient and control mice. After the treatment of 50 mg/d IMQ for six days, Zn-insufficient mice developed severer symptoms (Fig 7A) and higher PASI scores (Fig 7B), and produced more inflammatory factors than control mice (S7D Fig). Moreover, intranasal administration of zinc chloride (2 mg Zn/kg) before IMQ treatment relieved psoriasis symptoms (Figs 7C, 7D and S7E). Additionally, we established a Zn-rich mouse model using two months old APP/PS1 mice, which is a transgenic mouse model for Alzheimer’s disease (AD) [42]. Previous studies have shown that NLRP3 inflammasome activation drove Tau pathology to induce AD [43], so zinc supplementation may alleviate AD by inhibiting NLRP3. Two groups of mice were fed *ad libitum* with the standard diet (140 mg Zn/kg) or Zn-rich diet (700 mg Zn/kg) for four months. The growth of Zn-rich mice was unaffected during this period of feeding (S7F Fig). Zinc, but not copper and manganese, was increased in the brains of Zn-rich APP/PS1 mice (S7G and S7H Fig). Then, mouse brains were extracted and analyzed. We found that Tau phosphorylation was greatly suppressed, accompanied with the impaired NLRP3 activation in the brains of Zn-rich mice (Fig 7E), and the axons of Zn-rich mice were also significantly more than that of control mice (Fig 7F and 7G), indicative of relieved symptoms of AD. Furthermore, the spatial memory of Zn-rich mice was also significantly improved in Morris water maze test (Fig 7H and 7I), which suggested relieved symptoms of AD. Collectively, we found that zinc insufficiency aggravated psoriasis and zinc supplementation alleviated Alzheimer’s disease in mice, suggesting that zinc homeostasis regulates inflammasome-related autoimmune diseases.

## Discussion

As the second most abundant trace element for human, zinc takes part in various important physiological activities as many enzymes require Zn^2+^. However, its functions in innate immunity remain undefined. In this work, we found that Zinc transporter 1 (ZnT1) plays a critical role in controlling inflammasome activation and antiviral activation, specifically the cGAS-STING pathway. Although previous studies have shown that Zn^2+^ promotes dsDNA-induced cGAS phase separation [44], there is no type I-IFN induction in cells after Zn^2+^ treatment, which is consistent with our previous results. Therefore, increased IFN-β release in *SLC30A1*^−/−^ THP-1 cells was unlikely related to the direct activation of cGAS-STING pathway by Zn^2+^. *SLC30A1* deficiency blocked NLRP3 and NLRC4 inflammasome activation without affecting NF-κB signaling pathway. As the major carrier protein for zinc export out of cells, ZnT1 deficiency causes increased intracellular zinc content. The physiological concentration of zinc in human blood is around 50–100 μM [45]. We observed that both NLRP3 and NLRC4 inflammasome activation in THP-1 cells, a cell line derived from peripheral blood, was completely inhibited when the medium Zn^2+^ level reached 100 μM. Thus, the concentrations of Zn^2+^ required to inhibit inflammasomes appeared to be within the physiological range. We demonstrated that Zn^2+^ instead of other divalent metal cations bound inflammatory caspases at a conserved His-Cys-Cys triad to inhibit their enzymatic activity rather than affect ASC oligomerization, thus impairing the activation of multiple inflammasomes. Due to the critical role of caspases in inflammasome activation, zinc could be regarded as a broad-spectrum inflammasome inhibitor.

Inflammasome hyperactivation causes many devastating autoimmune diseases (e.g., gout, type II diabetes, rheumatoid arthritis, neurodegenerative diseases, Muckle-Wells syndrome and FCAS1) [46,47]. Common treatments for these diseases use anti-IL-1β monoclonal antibodies or the corresponding receptor antagonists [48,49]. However, inflammasome activation also releases IL-18 and many other danger- or damage-associated molecular patterns caused by pyroptotic cell death, all of which are pathogenic [50,51]. Thus, treatments that target upstream inflammasome components like caspases should be more effective [52–54]. Psoriasis is an autoimmune disease with high caspase-1 activity in psoriatic epidermis. There are many genetic studies supporting the role of NLRP3 in psoriasis susceptibility in patients [55–57]. Moreover, NLRP3 is highly expressed in the monocytes of patients with inflammatory dermatosis [58], and IL-1β secretion was detected in both human and mouse skin [59]. Abnormally activated NLRP3 triggers the release of IL-1β and IL-18. IL-1β promotes the differentiation of Th17 cells to secrete IFN-γ [60], and IL-18 enhances IFN-γ-induced production of C-X-C motif chemokine 9 (CXCL9), CXCL10 and CXCL11 [61], which migrate from dermis to epidermis causing chronic inflammatory activation of psoriasis. Unfortunately, psoriasis still lacks effective treatments. Although targeting NLRP3 is a promising way, only a few inhibitors of NLRP3 inflammasome are currently available in clinical [62]. Similarly, neuroinflammation causes AD development and progression [63], in which amyloid-β (Aβ) activates NLRP3 to mediate tau pathology and drive AD [43,46]. Only a few drugs are currently approved to treat AD whereas plenty of drugs have failed in clinical trials [64]. We showed that zinc supplementation significantly relieved sepsis and psoriasis in mice. Moreover, using Zn-insufficient and Zn-rich mice, we found that zinc homeostasis plays an important role in the regulation of inflammasome-related autoimmune diseases and that zinc supplementation effectively alleviates AD in mice, demonstrated by both biochemical and behavioral experiments. Due to the embryonic lethality of *Slc30a1*-deficient mice [65], we cannot directly test the role of Slc30a1 *in vivo*. Additionally, other studies also showed that zinc provides neuroprotection and alleviates autoimmune diseases like Behçet’s disease [66,67]. Therefore, our results offer a potential therapeutic strategy for the treatment of many inflammasome-related diseases, including sepsis that ranks amongst the most common causes of death in hospitalized patients.

## Materials and methods

### Ethics statement

Experiments were performed in accordance with the National Institute of Health Guide for Care and Use of Laboratory Animals with the approval of the Beijing Municipal Science & Technology Commission (SYXK (JING) 2024–0049).

### Mice

Age- and sex-matched C57BL/6J mice were used for experiments. All mice were bred and kept under specific pathogen-free conditions in the Laboratory Animal Center of Peking University.

### Cells

HEK293T, HT1080-ISRE and iBMDM cells were cultured in DMEM supplemented with 10% fetal bovine serum (FBS), 5 μg/mL penicillin and 10 μg/mL streptomycin. THP-1 cells were cultured in RPMI-1640 supplemented with 10% FBS, 5 μg/mL penicillin and 10 μg/mL streptomycin. All cells were cultured at 37°C in an incubator with 5% CO_2_.

### Reagents and resources

All reagents and resources used in this study are listed in S1 Table.

### Generation of gene-knockout cells

The sgRNAs were designed according to the target gene sequence. Cas9-expressing cells were infected with lentivirus produced with pSin-sgRNA-IRES-mCherry and sorted by FACS into 96-well plates. After about two weeks, single-cell clones were identified by sequencing. All sgRNAs used in this study are listed in S2 Table.

### Plasmid construction

The target DNA was amplified by PCR with a pair of primers, and the PCR product was digested with the corresponding restriction endonucleases. Then, the target DNA was connected to the eukaryotic or prokaryotic expression vector by T_4_ DNA ligase. All primers used in this study are listed in S3 Table.

### ASC oligomerization detection

After stimulation as indicated, cells were collected and lysed in a buffer containing 1% NP-40, 20 mM HEPES (pH 7.4), 150 mM KCl and some cocktail for 45 min on ice. The supernatants were removed by centrifugation at 6,000 g for 10 min, and the precipitation was washed by phosphate buffered saline (PBS) for two times. Then, the samples were supplemented with PBS containing DSS (2 mM) and applied to immunoblotting after 30 min at room temperature.

### Cecal ligation and puncture

Eight-week-old male and female mice were used for experiments. After the intraperitoneal injection of tribromoethanol (200 mg/kg body weight) for anesthesia, a laparotomy was performed. The cecum was mobilized from abdominal cavity through an incision about 1 cm, and ligated below the cecal valve using 3–0 silk. Then, the cecum was punctured 3 times with a 25G needle and some intestinal contents were squeezed out. Finally, the cecum was placed back and the abdominal cavity was closed by a 4–0 silk suture. No antibiotics and analgesics were used during the operation.

### Cell viability analysis

After the treatment as indicated, the cell viability was measured using a MTT (3-(4,5-dimethylthiazol-2-yl)-2,5-diphenyltetrazolium bromide) assay by the MTT Kit (Invitrogen, Cat# V13154) following the manufacturer’s instructions.

### ELISA

After the treatment as indicated, blood was harvested from the tail vein of mice. The sera were separated by centrifugation at 20,000 g for 15 min and applied to ELISA. Human IL-1β, TNF-α in cell supernatants and mouse IL-1β, IL-18, IL-6, TNF-α in sera were measured by the ELISA Kit (MultiSciences, Cat# EK101B, Cat# EK182, Cat# EK201B, Cat# EK218, Cat# EK206, Cat# EK282) following the manufacturer’s instructions.

### Genome-wide CRISPR-Cas9-mediated screen

A pooled human sgRNA library targeting 19,114 human genes was purchased from Addgene (Brunello, Cat# 73178), which was amplified following the protocol provided by the manufacturer [68]. For the CRISPR-Cas9-mediated screen, a total of 4×10^8^ pTRE3G-NLRP3 Tet-on THP-1 cells stably expressing Cas9 were transduced with the sgRNA lentiviral library at a MOI of 0.3. After 5 days of selection with puromycin (2 μg/mL), half of the puromycin-resistant cells were as the control group and the other half were as the experimental group. The cells in the experimental group were treated with Dox (100 ng/mL) for 8 h, and then the surviving cells were washed and cultured in fresh medium to recover several days to start next selection. After three rounds of selection, the surviving cells were collected to extract genomic DNA. The sgRNA-coding regions integrated into the chromosomes were amplified by PCR with primers lentiGP-1_F (AATGGACTATCATATGCTTACCGTAACTTG AAAGTATTTCG) and lentiGP-3_R (ATGAATACTGCCATTTGTCTCAAGATCTA GTTACGC). PCR products were subjected to electrophoresis and purification, followed by next-generation sequencing (NGS) analysis. Genes targeted by sgRNAs were ranked on the basis of the number of unique sgRNAs.

### Glycine silver staining

Mouse brain sections of 10 μm thickness were stained with silver by the glycine silver staining Kit (Servicebio, Cat# G1052) following the manufacturer’s instructions.

### Immunoblotting

Cells were washed with PBS and lysed in lysis buffer (20 mM HEPES (pH 7.4), 150 mM NaCl, 1.5 mM MgCl_2_, 12.5 mM β-glycerophosphate, 2 mM EGTA, 10 mM NaF, 1 mM Na_3_VO_4_, 0.5% Triton X-100, 2 mM DTT, 1 mM PMSF) for 30 min on ice. After the centrifugation at 20,000 g for 15 min, the supernatants were mixed with equivalent SDS loading buffer (100 mM Tris-HCl (pH 6.8), 4% SDS, 20% glycerol, 0.2 M DTT, 0.2% bromophenol blue) and boiled at 100°C for 10 min. Culture supernatants (Opti-MEM) were precipitated by adding an equal volume of methanol and 0.25 volumes of chloroform. After the centrifugation at 20,000 g for 15 min, the upper phase was discarded and fresh methanol was added to the interphase. The mixture was applied to centrifugation, and then the protein pellet was dried at 55°C for 5 min. Finally, the protein pellet was boiled at 100°C for 10 min in SDS loading buffer. The cells or supernatants samples were separated on 10% or 12% SDS-PAGE gels in running buffer (3 g/L Tris, 28 g/L Glycine, 1 g/L SDS) and electroblotted onto nitrocellulose (NC) membranes in transfer buffer (5.8 g/L Tris, 2.9 g/L Glycine, 10% methanol (v/v)). The membranes were blocked for 30 min in 5% skimmed milk in Tris-buffered saline Tween (TBST) buffer (3 g/L Tris, 8 g/L NaCl, 0.2 g/L KCl, 0.5% Tween-20 (v/v), pH 7.4). Then, the membranes were incubated with primary antibody of the target protein. After washing, the membranes were incubated with specific appropriate horseradish peroxidase-conjugated secondary antibody and visualized by Odyssey imaging system.

### Intracellular zinc content identified by TSQ

Cells were treated with TSQ (5 μg/mL) for 30 min at 37°C and then washed and cultured in fresh medium. The samples were applied to fluorescence microscopy and flow cytometry analysis. Microscope images were acquired on a Leica DMI4000 microscope using a 10 × or 40 × objective.

### *In vitro* cleavage assay

Recombinant human caspase-1 and GSDMD or pro-IL-1β were co-incubated with different divalent metal cations in cleavage buffer (50 mM HEPES (pH 7.4), 150 mM NaCl, 5 mM DTT) for 2 h at 37°C, and then the samples were applied to immunoblotting.

### Morris water maze test

Mice were trained in a round, water-filled tub in an environment rich with maze cues. Each mouse was given four trials per day for four consecutive days with a 15 min intertrial interval. The maximum trial length was 60 s, and if mice did not reach the platform, they were manually guided to it and stayed for 10 s. After four days of training, the platform was removed, and a probe test was carried out to quantify the percentage of time spent in the quadrant that previously contained the platform in 60 s.

### Protein expression and purification

Human GSDMD, pro-IL-1β, caspase-1 and variants full length were cloned into pET-21b vector, and confirmed by sequencing. The plasmids were transformed into E. coli BL21 (DE3) and induced with 1 mM isopropyl-β-D-thiogalactoside (IPTG) for 5 h at 37°C when the OD value was between 0.6–0.8. Protein was purified by a Ni-NTA His affinity column.

### Type I IFN bioassay

The production of type I IFNs in cell supernatants was measured as previously described [69]. Briefly, HT1080-ISRE cells which have an IFN-stimulated response element were seeded into 96-well plates and incubated with cell supernatants for 4 h. Then, cells were lysed and measured by the luciferase reporter assay system (Promega, Cat# E1500).

### Viral infection

For cell stimulation, cells were infected with each type of virus at a MOI of 0.1 for 1 h and then washed and cultured in fresh medium. For lentiviral infection, lentivirus was produced by co-transfection of the target plasmid and two packaging plasmids, psPAX2 and pMD2.G, into HEK293T cells. After 6–8 hours, the medium was replaced with DMEM supplemented with 20% FBS. The lentivirus-containing supernatants were harvested 48 h after transfection. Cells were co-cultured with the supernatants and polybrene (4 μg/mL) for 8 h, and then washed and cultured in fresh medium. Two days later, cells were selected for the transgene characteristics.

### Zinc-binding stoichiometry identified by ICP-MS

Purified caspase-1 or variants were incubated with an overdose of ZnCl_2_ in the buffer containing 50 mM HEPES (pH 7.4), 150 mM NaCl and 5 mM DTT for 1 h at 4°C. Unbound zinc was removed by adding Chelex-100 (0.1 g/mL) to the samples for 1 h at 4°C, mixing every 15 min. After the centrifugation at 20,000 g for 15 min, zinc content in the supernatants was quantified by inductively coupled plasma mass spectrometry (ICP-MS).

### Zinc-binding stoichiometry identified by zincon

A 1 mL solution containing 10 μM purified caspase-1 or variants, 50 μM zincon, 50 mM HEPES (pH 7.4), 150 mM NaCl and 5 mM DTT was titrated by ZnCl_2_ at room temperature, and the absorbance at 620 nm was recorded after each addition. The normalized absorbance was plotted against the molar equivalents of Zn added per caspase-1 monomer.

### Statistical analysis

Student’s t-test, ANOVA test and Mantel-Cox test were used to analyze data. Data are shown as mean ± SD. N.S., not significant, p > 0.05; *p < 0.05; **p < 0.01; ***p < 0.001; ****p < 0.0001.

## Supporting information

S1 Fig pTRE3G-NLRP3 Tet-on THP-1 cells shows nice response to Dox in a Dox concentration-dependent manner. (A) Immunoblotting analysis of the indicated proteins in pTRE3G-NLRP3 Tet-on THP-1 cells treated with Dox (0 ng/mL, 100 ng/mL and 500 ng/mL) for 8 h. (B) Cell viability analysis of pTRE3G-NLRP3 Tet-on THP-1 cells treated with Dox (0 ng/mL, 100 ng/mL and 500 ng/mL) for 8 h. Data are the mean ± SD. ANOVA test was used to analyze data. ****p < 0.0001. (C and D) IL-1β (C) and TNF-α (D) concentration of cell supernatants from pTRE3G-NLRP3 Tet-on THP-1 cells pretreated with LPS (1 μg/mL) or saline for 3 h, followed by Dox (100 ng/mL) for 8 h. Data are the mean ± SD. ANOVA test was used to analyze data. N.S., not significant; ***p < 0.001; ****p < 0.0001. (E) The enrichment of four sgRNAs of *SLC30A1* in the genome-wide CRISPR-Cas9-mediated screen.
(TIF)

S2 Fig *SLC30A1* deficiency enhances DNA virus-induced innate immune response. (A) Immunoblotting analysis of the indicated proteins in WT and *SLC30A1*^*−/−*^ THP-1 cells. (B) Genotyping of *SLC30A1*^*−/−*^ THP-1 cells. (C) Immunoblotting analysis of the indicated proteins in WT, *SLC30A1*^*−/−*^ and SLC30A1 re-expression THP-1 cells infected with VACV (MOI = 0.1) or HSV-1 (MOI = 0.1) for the indicated times. (D) Type I interferon assay of cell supernatants from WT, *SLC30A1*^*−/−*^ and SLC30A1 re-expression THP-1 cells infected with VACV (MOI = 0.1) or HSV-1 (MOI = 0.1) for 12 h. Data are the mean ± SD.
(TIF)

S3 Fig *SLC30A1* deficiency increases the zinc content in THP-1 cells. (A) The structural formula of TSQ (C_17_H_16_N_2_O_3_S) fluorescent probe. (B) The fluorescence intensity of WT, *SLC30A1*^*−/−*^ and SLC30A1 re-expression THP-1 cells treated with TSQ (5 μg/mL) for 30 min. Data are the mean ± SD. ANOVA test was used to analyze data. ****p < 0.0001. (C) Zinc content of WT, *SLC30A1*^*−/−*^ and SLC30A1 re-expression THP-1 cells identified by ICP-MS. Data are the mean ± SD. ANOVA test was used to analyze data. ***p < 0.001.
(TIF)

S4 Fig Zinc impairs inflammasome activation with normal NF-κB. (A) Immunoblotting analysis of the indicated proteins in pTRE3G-NLRP3 Tet-on THP-1 cells pretreated with ZnCl_2_ (50 μM), CuCl_2_ (50 μM), FeCl_2_ (100 μM) or MnCl_2_ (10 μM) for 1 h, followed by Dox (100 ng/mL) for 8 h. C, Con. (B) Immunoblotting analysis of the indicated proteins in WT THP-1 cells pretreated with the indicated concentration of ZnCl_2_ for 1 h, followed by LPS (1 μg/mL) for the indicated times.
(TIF)

S5 Fig Zinc binds caspases at a conserved His-Cys-Cys triad. (A) Schematic illustration of caspase-1 autoprocessing. (B) Zn^2+^-binding site of caspase-9 (marked in orange). The protein structure data was downloaded from PDB (1JXQ) and opened by ChimeraX. (C) Cell viability analysis of WT, *SLC30A1*^−/−^ and *CASP9*^−/−^ THP-1 cells pretreated with ZnCl_2_ (100 μM) or saline for 1 h, followed by ABT-263 (20 μM) for 24 h or LPS (1 μg/mL) for 3 h and nigericin (5 μM) for 1 h. Data are the mean ± SD. ANOVA test was used to analyze data. *p < 0.05; ****p < 0.0001. (D) Zn^2+^-binding stoichiometry of caspase-1 and variants identified by zincon. (E) Sequence alignment of caspase-4/5 (human) and caspase-11 (mouse). Protein sequences were obtained from UniProt. Casp4_HUMAN: P49662; Casp5_HUMAN: P51878; Casp11_MOUSE: P70343.
(TIF)

S6 Fig *Slc30a1* deficiency increases the zinc content in iBMDM cells. (A) Genotyping of *Slc30a1*^*−/−*^ iBMDM cells. (B) Fluorescence microscopy of WT and *Slc30a1*^*−/−*^ iBMDM cells treated with TSQ (5 μg/mL) for 30 min. The intracellular zinc content is characterized by blue fluorescence. Scale bar, 100 μm. (C) The fluorescence intensity of WT and *Slc30a1*^*−/−*^ iBMDM cells treated with TSQ (5 μg/mL) for 30 min. Data are the mean ± SD. Student’s t-test was used to analyze data. ***p < 0.001. (D) Flow cytometry analysis of WT and *Slc30a1*^*−/−*^ iBMDM cells treated with TSQ (5 μg/mL) for 30 min. P4 represents positive cell population. (E) Zinc content of WT and *Slc30a1*^*−/−*^ iBMDM cells identified by ICP-MS. Data are the mean ± SD. Student’s t-test was used to analyze data. **p < 0.01. (F) Survival of the mice (n = 7) pretreated (i.v.) with saline or ZnCl_2_ (2 mg Zn/kg) for 24 h, followed by cecal ligation and puncture operation. Survival curves were compared using Mantel-Cox test.
(TIF)

S7 Fig The level of trace elements and cytokines of Zn-insufficient and Zn-rich mice. (A) Body weight of the control (Con) and Zn-insufficient (-Zn) mice. Data are the mean ± SD (n = 5). (B and C) Zn (B), Cu and Mn (C) content in the indicated organs of the control (Con) and Zn-insufficient (-Zn) mice identified by ICP-MS. Data are the mean ± SD (n = 3). (D) IL-1β, IL-18, IL-6 and TNF-α concentration of sera from the control (Con) and Zn-insufficient (-Zn) mice treated with IMQ (50 mg/d). Blood samples were collected at 6 days after the treatment. Data are the mean ± SD (n = 4). (E) IL-1β, IL-18, IL-6 and TNF-α concentration of sera from the control (Con) and Zn-rich (+Zn) mice treated with IMQ (60 mg/d). Blood samples were collected at 6 days after the treatment. Data are the mean ± SD (n = 4). (F) Body weight of the control (Con) and Zn-rich (+Zn) mice. Data are the mean ± SD (n = 6). (G and H) Zn (G), Cu and Mn (H) content in the indicated organ of the control (Con) and Zn-rich (+Zn) mice identified by ICP-MS. Data are the mean ± SD (n = 3). Student’s t-test was used to analyze data. N.S., not significant, p > 0.05; *p < 0.05; **p < 0.01.
(TIF)

S1 Table Reagents and resources used in this study. (DOCX)

S2 Table SgRNAs used in this study. (DOCX)

S3 Table Primers used in this study. (DOCX)
