# Supplementary figures and images for: Zinc homeostasis regulates caspase activity and inflammasome activation
Source: PLoS Pathog. 2024 Dec 17;20(12):e1012805. doi: 10.1371/journal.ppat.1012805 (PMC11687882; doi:10.1371/journal.ppat.1012805)

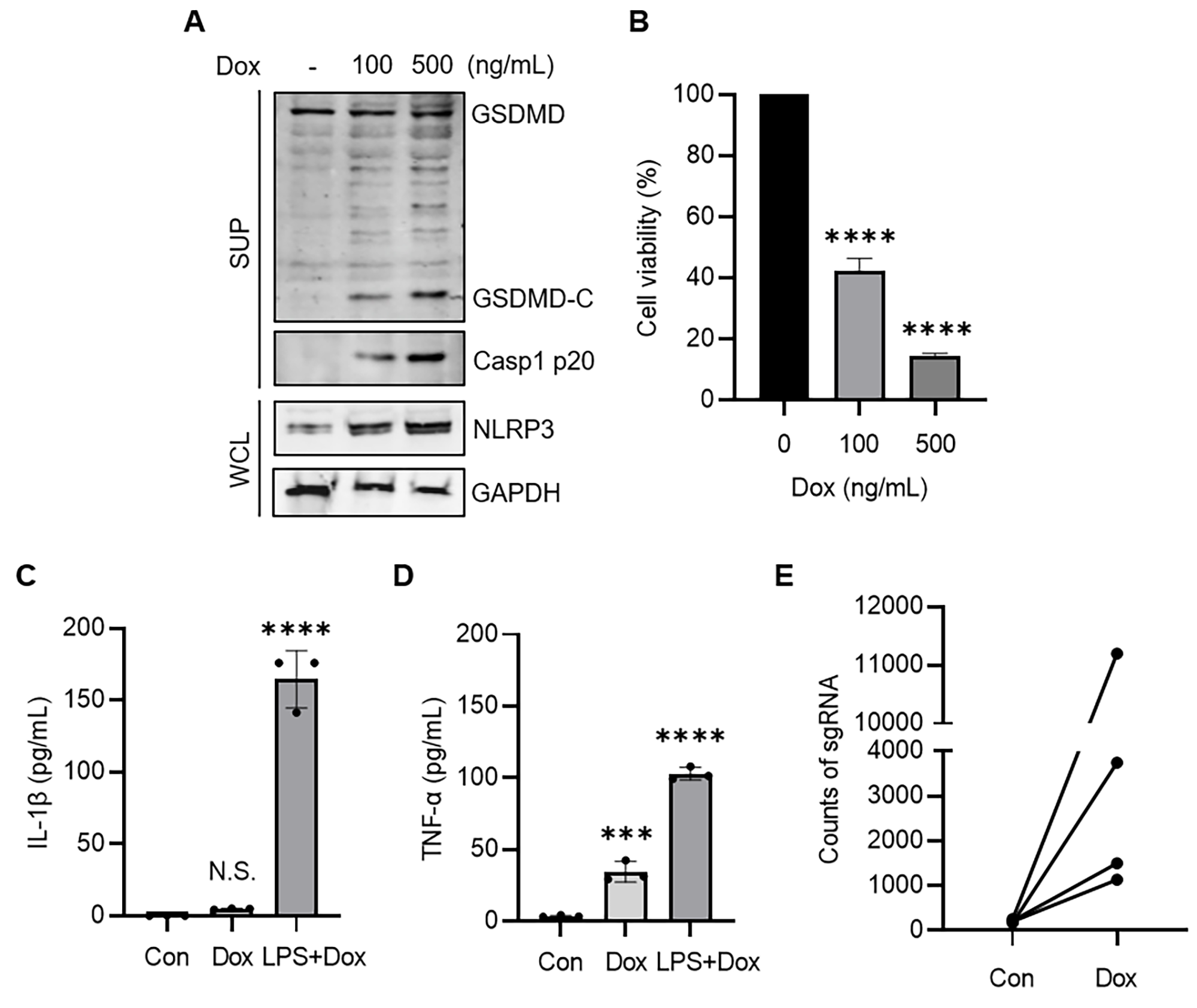

Supplement: S1 Fig — (A) Immunoblotting analysis of the indicated proteins in pTRE3G-NLRP3 Tet-on THP-1 cells treated with Dox (0 ng/mL, 100 ng/mL and 500 ng/mL) for 8 h. (B) Cell viability analysis of pTRE3G-NLRP3 Tet-on THP-1 cells treated with Dox (0 ng/mL, 100 ng/mL and 500 ng/mL) for 8 h. Data are the mean ± SD. ANOVA test was used to analyze data. ****p < 0.0001. (C and D) IL-1β (C) and TNF-α (D) concentration of cell supernatants from pTRE3G-NLRP3 Tet-on THP-1 cells pretreated with LPS (1 μg/mL) or saline for 3 h, followed by Dox (100 ng/mL) for 8 h. Data are the mean ± SD. ANOVA test was used to analyze data. N.S., not significant; ***p < 0.001; ****p < 0.0001. (E) The enrichment of four sgRNAs of SLC30A1 in the genome-wide CRISPR-Cas9-mediated screen. (TIF) [file ppat.1012805.s001.tif]

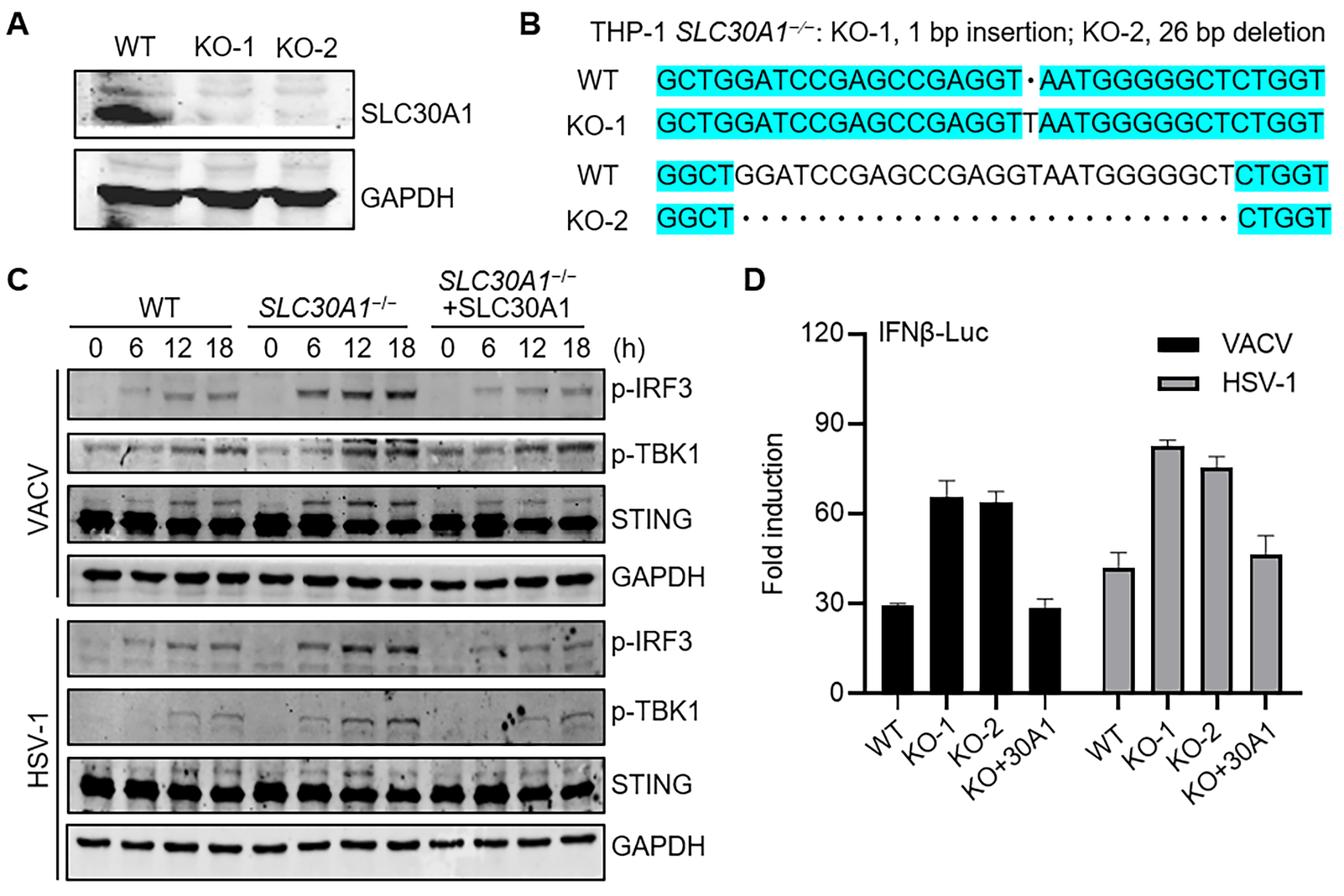

Supplement: S2 Fig — (A) Immunoblotting analysis of the indicated proteins in WT and SLC30A1−/− THP-1 cells. (B) Genotyping of SLC30A1−/− THP-1 cells. (C) Immunoblotting analysis of the indicated proteins in WT, SLC30A1−/− and SLC30A1 re-expression THP-1 cells infected with VACV (MOI = 0.1) or HSV-1 (MOI = 0.1) for the indicated times. (D) Type I interferon assay of cell supernatants from WT, SLC30A1−/− and SLC30A1 re-expression THP-1 cells infected with VACV (MOI = 0.1) or HSV-1 (MOI = 0.1) for 12 h. Data are the mean ± SD. (TIF) [file ppat.1012805.s002.tif]

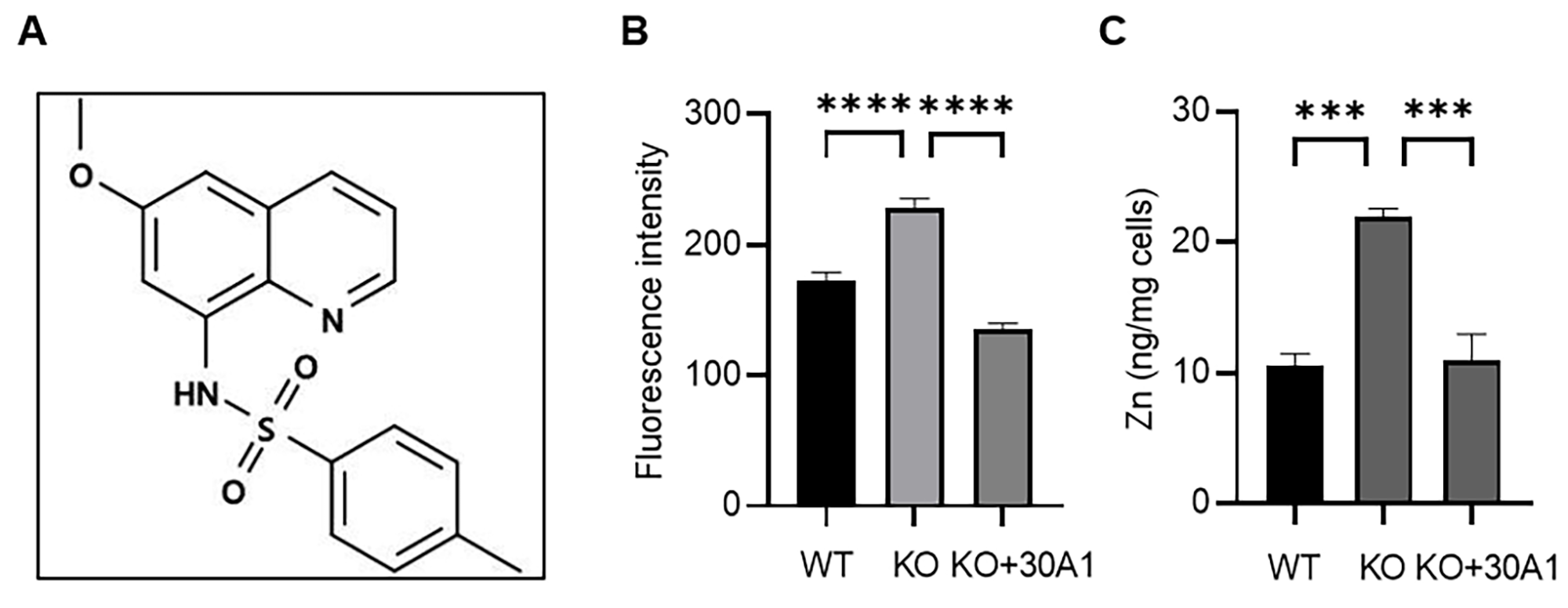

Supplement: S3 Fig — (A) The structural formula of TSQ (C17H16N2O3S) fluorescent probe. (B) The fluorescence intensity of WT, SLC30A1−/− and SLC30A1 re-expression THP-1 cells treated with TSQ (5 μg/mL) for 30 min. Data are the mean ± SD. ANOVA test was used to analyze data. ****p < 0.0001. (C) Zinc content of WT, SLC30A1−/− and SLC30A1 re-expression THP-1 cells identified by ICP-MS. Data are the mean ± SD. ANOVA test was used to analyze data. ***p < 0.001. (TIF) [file ppat.1012805.s003.tif]

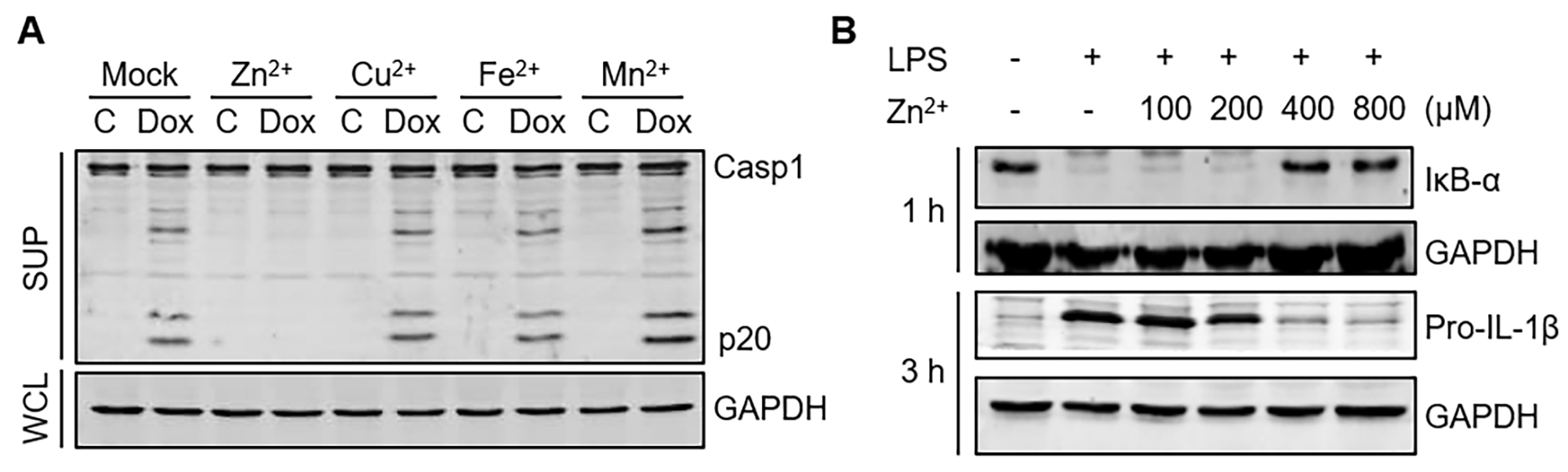

Supplement: S4 Fig — (A) Immunoblotting analysis of the indicated proteins in pTRE3G-NLRP3 Tet-on THP-1 cells pretreated with ZnCl2 (50 μM), CuCl2 (50 μM), FeCl2 (100 μM) or MnCl2 (10 μM) for 1 h, followed by Dox (100 ng/mL) for 8 h. C, Con. (B) Immunoblotting analysis of the indicated proteins in WT THP-1 cells pretreated with the indicated concentration of ZnCl2 for 1 h, followed by LPS (1 μg/mL) for the indicated times. (TIF) [file ppat.1012805.s004.tif]

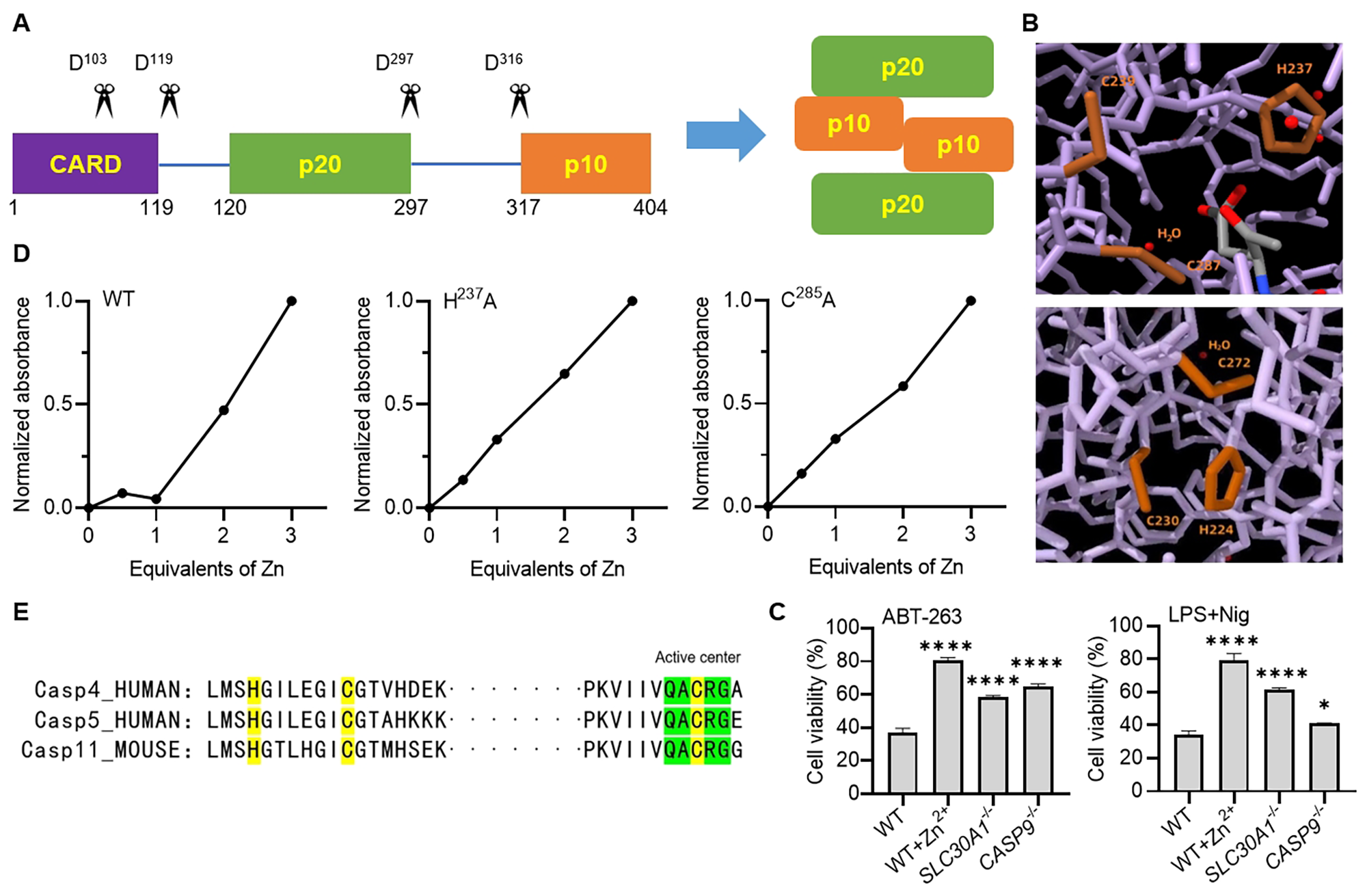

Supplement: S5 Fig — (A) Schematic illustration of caspase-1 autoprocessing. (B) Zn2+-binding site of caspase-9 (marked in orange). The protein structure data was downloaded from PDB (1JXQ) and opened by ChimeraX. (C) Cell viability analysis of WT, SLC30A1−/− and CASP9−/− THP-1 cells pretreated with ZnCl2 (100 μM) or saline for 1 h, followed by ABT-263 (20 μM) for 24 h or LPS (1 μg/mL) for 3 h and nigericin (5 μM) for 1 h. Data are the mean ± SD. ANOVA test was used to analyze data. *p < 0.05; ****p < 0.0001. (D) Zn2+-binding stoichiometry of caspase-1 and variants identified by zincon. (E) Sequence alignment of caspase-4/5 (human) and caspase-11 (mouse). Protein sequences were obtained from UniProt. Casp4_HUMAN: P49662; Casp5_HUMAN: P51878; Casp11_MOUSE: P70343. (TIF) [file ppat.1012805.s005.tif]

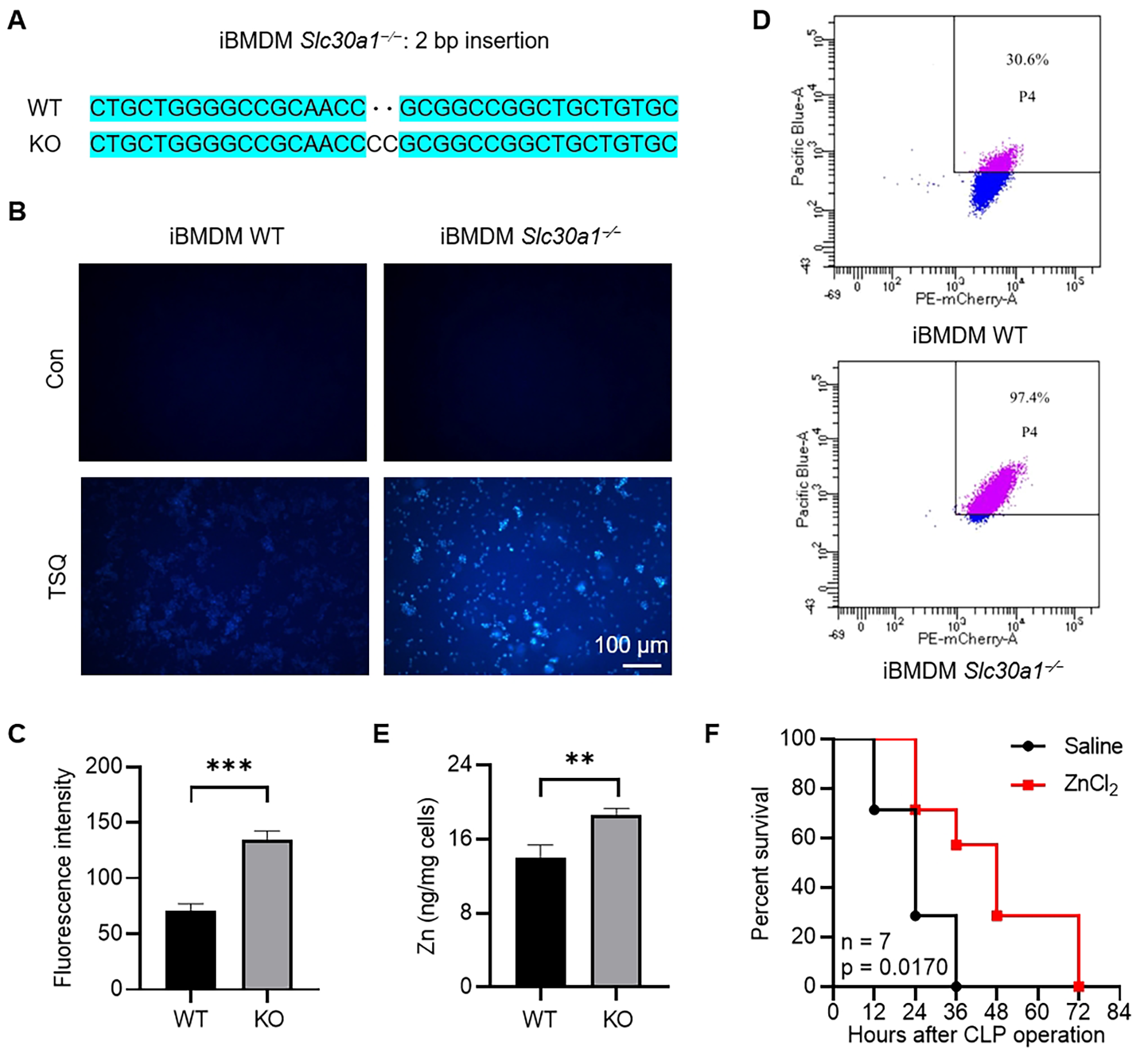

Supplement: S6 Fig — (A) Genotyping of Slc30a1−/− iBMDM cells. (B) Fluorescence microscopy of WT and Slc30a1−/− iBMDM cells treated with TSQ (5 μg/mL) for 30 min. The intracellular zinc content is characterized by blue fluorescence. Scale bar, 100 μm. (C) The fluorescence intensity of WT and Slc30a1−/− iBMDM cells treated with TSQ (5 μg/mL) for 30 min. Data are the mean ± SD. Student’s t-test was used to analyze data. ***p < 0.001. (D) Flow cytometry analysis of WT and Slc30a1−/− iBMDM cells treated with TSQ (5 μg/mL) for 30 min. P4 represents positive cell population. (E) Zinc content of WT and Slc30a1−/− iBMDM cells identified by ICP-MS. Data are the mean ± SD. Student’s t-test was used to analyze data. **p < 0.01. (F) Survival of the mice (n = 7) pretreated (i.v.) with saline or ZnCl2 (2 mg Zn/kg) for 24 h, followed by cecal ligation and puncture operation. Survival curves were compared using Mantel-Cox test. (TIF) [file ppat.1012805.s006.tif]

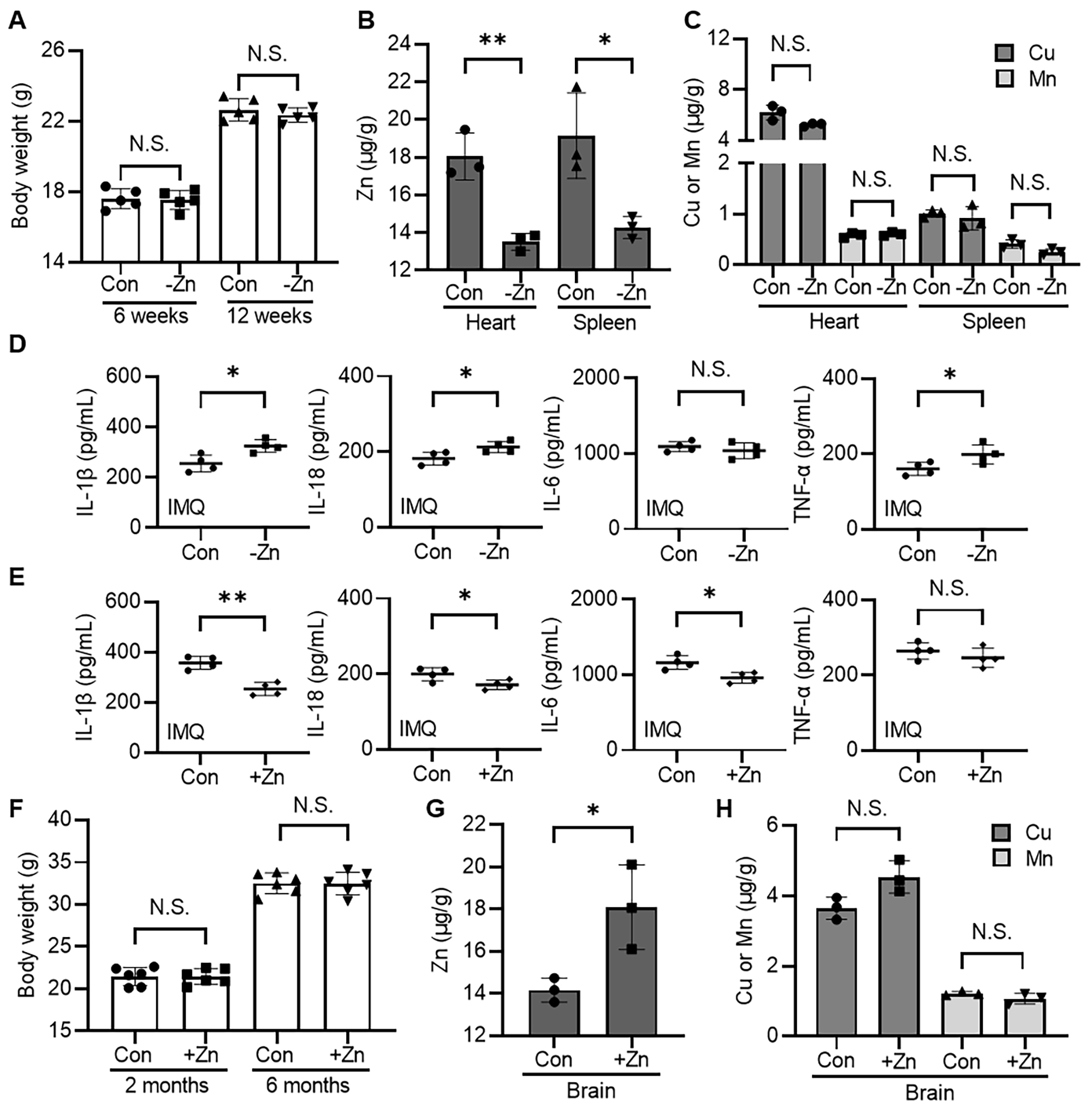

Supplement: S7 Fig — (A) Body weight of the control (Con) and Zn-insufficient (-Zn) mice. Data are the mean ± SD (n = 5). (B and C) Zn (B), Cu and Mn (C) content in the indicated organs of the control (Con) and Zn-insufficient (-Zn) mice identified by ICP-MS. Data are the mean ± SD (n = 3). (D) IL-1β, IL-18, IL-6 and TNF-α concentration of sera from the control (Con) and Zn-insufficient (-Zn) mice treated with IMQ (50 mg/d). Blood samples were collected at 6 days after the treatment. Data are the mean ± SD (n = 4). (E) IL-1β, IL-18, IL-6 and TNF-α concentration of sera from the control (Con) and Zn-rich (+Zn) mice treated with IMQ (60 mg/d). Blood samples were collected at 6 days after the treatment. Data are the mean ± SD (n = 4). (F) Body weight of the control (Con) and Zn-rich (+Zn) mice. Data are the mean ± SD (n = 6). (G and H) Zn (G), Cu and Mn (H) content in the indicated organ of the control (Con) and Zn-rich (+Zn) mice identified by ICP-MS. Data are the mean ± SD (n = 3). Student’s t-test was used to analyze data. N.S., not significant, p > 0.05; *p < 0.05; **p < 0.01. (TIF) [file ppat.1012805.s007.tif]
