# Supplementary material for: Zinc homeostasis regulates caspase activity and inflammasome activation
Source: PLoS Pathog. 2024 Dec 17;20(12):e1012805. doi: 10.1371/journal.ppat.1012805 (PMC11687882; doi:10.1371/journal.ppat.1012805)
Supplement: S1 Table — (DOCX) [file ppat.1012805.s008.docx]

**S1 Table: Reagents and resources used in this study.**

| **Reagents and Resources** | **Source** | **Identifier** |
| --- | --- | --- |
| **Antibodies** |  |  |
| Mouse monoclonal anti-Flag | Sigma-Aldrich | Cat# F3165 |
| Mouse polyclonal anti-hcaspase-1 | This paper | N/A |
| Rabbit polyclonal anti-hIL-1β | This paper | N/A |
| Rabbit polyclonal anti-hGSDMD | This paper | N/A |
| Mouse polyclonal anti-hNLRP3 | This paper | N/A |
| Mouse polyclonal anti-hASC | This paper | N/A |
| Mouse monoclonal anti-hIκB-α | CST | Cat# 4814 |
| Rabbit monoclonal anti-hp-TBK1 (Ser172) | Abcam | Cat# ab109272 |
| Rabbit monoclonal anti-hp-IRF3 (Ser396) | CST | Cat# 4947 |
| Rabbit polyclonal anti-hSTING | This paper | N/A |
| Rabbit polyclonal anti-hSLC30A1 | Sigma-Aldrich | Cat# A78431 |
| Rabbit polyclonal anti-hGAPDH | Santa Cruz | Cat# sc-25778 |
| Mouse polyclonal anti-hLamin A/C | This paper | N/A |
| Rabbit polyclonal anti-mcaspase-1 | This paper | N/A |
| Rabbit polyclonal anti-mIL-1β | This paper | N/A |
| Rabbit polyclonal anti-mGSDMD | This paper | N/A |
| Rabbit polyclonal anti-mGAPDH | This paper | N/A |
| Mouse monoclonal anti-mTau (Tau-5) | Santa Cruz | Cat# sc-58860 |
| Rabbit polyclonal anti-mp-Tau (Ser396) | Invitrogen | Cat# 44-752G |
| **Bacterial and Virus Strains** |  |  |
| E. coli BL21 (DE3) | Transgene | CD-601 |
| E. coli DH5α | Biomed | BC102 |
| *Salmonella Typhimurium* | Dr. Feng Shao | N/A |
| Vaccinia virus | Dr. Meilin Jin | N/A |
| Herpes simplex virus-1 | Dr. Hongbing Shu | N/A |
| **Chemicals** |  |  |
| TSQ | AAT Bioquest | Cat# 21254 |
| Polyethyleneimine | Polyscience | Cat# 23966 |
| Polybrene | Aladdin | CAS: 28728-55-4 |
| Puromycin | Aladdin | CAS: 53-79-2 |
| Doxycycline | Aladdin | CAS: 564-25-0 |
| Lipopolysaccharide | Aladdin | CAS: 93572-42-0 |
| Pam2CSK4 | Aladdin | CAS: 868247-72-7 |
| Nigericin | Aladdin | CAS: 28380-24-7 |
| Zincon | Aladdin | CAS: 62625-22-3 |
| VX-765 | MCE | CAS: 273404-37-8 |
| z-VAD-FMK | MCE | CAS: 161401-82-7 |
| Imiquimod | MED-SHINE | CAS: 99011-02-6 |
| Chelex-100 | BIOFOUNT | CAS: 11139-85-8 |
| ABT-263 | Selleck | CAS: 923564-51-6 |
| ATP | Sigma-Aldrich | Cat# A7699 |
| IPTG | Sigma-Aldrich | Cat# I6758 |
| NaCl | Sigma-Aldrich | Cat# S9888 |
| ZnCl_2_ | Sigma-Aldrich | Cat# Z0152 |
| FeCl_2_ | Sigma-Aldrich | Cat# 372870 |
| CuCl_2_🞄2H_2_O | Sigma-Aldrich | Cat# C3279 |
| MnCl_2_🞄4H_2_O | Sigma-Aldrich | Cat# M5005 |
| Zn-deficient mouse diet | Shuyu Biology | N/A |
| Zn-rich mouse diet | Shuyu Biology | N/A |
| **Critical Commercial Assays** |  |  |
| Luciferase Reporter Assay System | Promega | Cat# E1500 |
| Vybrant MTT Cell Proliferation Assay Kit | Invitrogen | Cat# V13154 |
| Glycine Silver Staining Kit | Servicebio | Cat# G1052 |
| Human IL-1β ELISA Kit | MultiSciences | Cat# EK101B |
| Human TNF-α ELISA Kit | MultiSciences | Cat# EK182 |
| Mouse IL-1β ELISA Kit | MultiSciences | Cat# EK201B |
| Mouse IL-18 ELISA Kit | MultiSciences | Cat# EK218 |
| Mouse IL-6 ELISA Kit | MultiSciences | Cat# EK206 |
| Mouse TNF-α ELISA Kit | MultiSciences | Cat# EK282 |
| **Experimental Models: Cell Lines** |  |  |
| Human: pTRE3G-NLRP3 Tet-on THP-1 cells | This paper | N/A |
| Human: HT1080-ISRE cells | Dr. G. Stark | N/A |
| Human: HEK293T cells | ATCC | Cat# CRL-11268 |
| Human: THP-1 cells | ATCC | Cat# TIB-202 |
| Human: *SLC30A1^−⁄−^* THP-1 cells | This paper | N/A |
| Human: *CASP1^−⁄−^* THP-1 cells | This paper | N/A |
| Human: *CASP9^−⁄−^* THP-1 cells | This paper | N/A |
| Mouse: iBMDM cells | Dr. K. Fitzgerald | N/A |
| Mouse: *Slc30a1^−⁄−^* iBMDM cells | This paper | N/A |
| **Experimental Models: Organisms/Strains** |  |  |
| C57BL/6J | Charles River | N/A |
| **Recombinant Plasmid** |  |  |
| Plasmid: pU6-sgRNA | Dr. Feng Zhang | N/A |
| Plasmid: pcDNA3.1-Cas9 | Dr. Feng Zhang | N/A |
| Plasmid: pLVX-Tet3G | Dr. Hong Wu | N/A |
| Plasmid: psPAX2 | Addgene | Cat# 12260 |
| Plasmid: pMD2.G | Addgene | Cat# 12259 |
| Plasmid: pTRE3G-NLRP3 | This paper | N/A |
| Plasmid: pET-21b-caspase-1 | This paper | N/A |
| Plasmid: pET-21b-caspase-1 (H^237^A) | This paper | N/A |
| Plasmid: pET-21b-caspase-1 (C^285^A) | This paper | N/A |
| Plasmid: pET-21b-GSDMD | This paper | N/A |
| Plasmid: pET-21b-pro-IL-1β | This paper | N/A |
| Plasmid: pSin-SLC30A1-Flag-IRES-Puro | This paper | N/A |
| Plasmid: pSin-BFP-ASC-IRES-Puro | This paper | N/A |
| **Software and Algorithms** |  |  |
| GraphPad Prism 8 | GraphPad | N/A |
| FlowJo 10.8.1 | FlowJo | N/A |
| ChimeraX 1.4 | ChimeraX | N/A |
| ImageJ 1.8.0 | ImageJ | N/A |
| **Other** |  |  |
| DMEM | Gibco | Cat# C22400500BT |
| RPMI-1640 | Gibco | Cat# C11995500BT |
| Opti-MEM | Gibco | Cat# 11058021 |
| PBS | Gibco | Cat# 10010023 |
| FBS | Gibco | Cat# 10099141 |
| Trypsin | Gibco | Cat# 25300120 |
| Penicillin-Streptomycin | Gibco | Cat# 15140122 |
