# Supplementary material for: Zinc homeostasis regulates caspase activity and inflammasome activation
Source: PLoS Pathog. 2024 Dec 17;20(12):e1012805. doi: 10.1371/journal.ppat.1012805 (PMC11687882; doi:10.1371/journal.ppat.1012805)
Supplement: S2 Table — (DOCX) [file ppat.1012805.s009.docx]

**S2 Table: SgRNAs used in this study.**

| **Target gene** | **Forward** | **Reverse** |
| --- | --- | --- |
| hSLC30A1 | TGGATCCGAGCCGAGGTAAT | ATTACCTCGGCTCGGATCCA |
| hSLC30A1 | GGATCCGAGCCGAGGTAATG | CATTACCTCGGCTCGGATCC |
| mSLC30A1 | ACAGCAGCCGGCCGCGGTTG | CAACCGCGGCCGGCTGCTGT |
