# Supplementary material for: Zinc homeostasis regulates caspase activity and inflammasome activation
Source: PLoS Pathog. 2024 Dec 17;20(12):e1012805. doi: 10.1371/journal.ppat.1012805 (PMC11687882; doi:10.1371/journal.ppat.1012805)
Supplement: S3 Table — (DOCX) [file ppat.1012805.s010.docx]

**S3 Table: Primers used in this study.**

| **Target DNA** | **Forward** | **Reverse** |
| --- | --- | --- |
| pTRE3G-NLRP3 | CAAGCTTGCGGTACCGCGGGCCCGGGATGGACTACAAAGACCATGA | CAGGTCGACTCTAGAGTCGCGGCCGCCTACCAAGAAGGCTCAAAGA |
| pET-21b-caspase-1 | ACGCGTCGACATGGCCGACAAGGTCCTGAA | ATAAGAATGCGGCCGCTTAATGTCCTGGGAAGAGGT |
| pET-21b-caspase-1 H^237^A | CTGGTGTTCATGTCTGCTGGTATTCGGGAAGG | CCTTCCCGAATACCAGCAGACATGAACACCAG |
| pET-21b-caspase-1 C^285^A | ATCATCATCCAGGCCGCCCGTGGTGACAGCCC | GGGCTGTCACCACGGGCGGCCTGGATGATGAT |
| pET-21b-GSDMD | ACGCGTCGACATGGGGTCGGCCTTTGAGCG | ATAAGAATGCGGCCGCCTAGTGGGGCTCCTGGCTCAGTCC |
| pET-21b-pro-IL-1β | ACGCGTCGACATGGCAGAAGTACCTGAGCT | ATAAGAATGCGGCCGCTTAGGAAGACACAAATTGCATGGT |
| pSin-SLC30A1-Flag-IRES-Puro | CTAGGCTAGCATGGGGTGTTGGGGTCG | ACTCGTTAACCAAAGATGATTCAGGTTGTTTGT |
| pSin-BFP-ASC-IRES-Puro | CGACGCGTATGGGGCGCGCGCGCGACGC | ACGCGTCGTCAGCTCCGCTCCAGGTCCT |
